# Role of SUMO activating enzyme in cancer stem cell maintenance and self-renewal

**DOI:** 10.1038/ncomms12326

**Published:** 2016-07-28

**Authors:** Li Du, Yi-Jia Li, Marwan Fakih, Rebecca L. Wiatrek, Marjun Duldulao, Zhenbin Chen, Peiguo Chu, Julio Garcia-Aguilar, Yuan Chen

**Affiliations:** 1 Department of Molecular Medicine, Beckman Research Institute of City of Hope, Duarte, California 91010, USA; 2 Department of Medical Oncology, City of Hope National Medical Center, Duarte, California 91010, USA; 3 Department of Surgery, City of Hope National Medical Center, Duarte, California 91010, USA; 4 Department of Pathology, City of Hope National Medical Center, Duarte, California 91010, USA

## Abstract

Cancer stem cells (CSCs) have key roles in treatment resistance, tumour metastasis and relapse. Using colorectal cancer (CC) cell lines, patient-derived xenograft (PDX) tissues and patient tissues, here we report that CC CSCs, which resist chemoradiation, have higher SUMO activating enzyme (E1) and global SUMOylation levels than non-CSCs. Knockdown of SUMO E1 or SUMO conjugating enzyme (E2) inhibits CC CSC maintenance and self-renewal, while overexpression of SUMO E1 or E2 increases CC cell stemness. We found that SUMOylation regulates CSCs through Oct-1, a transcription factor for aldehyde dehydrogenases (ALDHs). ALDH activity is not only a marker for CSCs but also important in CSC biology. SUMO does not modify Oct-1 directly, but regulates the expression of TRIM21 that enhances Oct-1 ubiquitination and, consequently, reducing Oct-1 stability. In summary, our findings suggest that SUMOylation could be a target to inhibit CSCs and ultimately to reduce treatment resistance, tumour metastasis and relapse.

## Abstract (web summary)

Cancer stem cells (CSCs) have key roles in tumor initiation and metastasis. Here, the authors show that the SUMO E1 and global sumoylation levels are high in colorectal CSCs and that depletion of the catalytic subunit of the SUMO E1, SAE2, affects CSCs self-renewal through TRIM21-mediated degradation of the Oct1, a transcription factor for ALDH.

## Graphical abstract

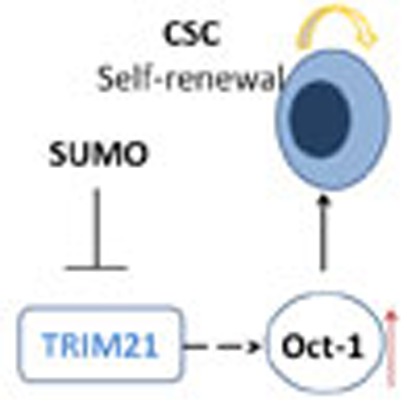

Cancer stem cells (CSC) exist in both blood cancers and solid tumours^1^,^2^,^3^, and present a major obstacle in cancer therapy^4^. These small populations of cells are capable of growing into new cancers^5^,^6^. In addition, CSCs often evade chemotherapy and radiation (chemoradiation), both of which typically target rapidly dividing non-CSCs. Furthermore, emerging evidence indicates that chemoradiation increases CSC populations^7^,^8^,^9^, either by eradicating non-CSCs or by inducing dedifferentiation of non-CSCs. CSCs then seed tumour regrowth at the original or a distant site, resulting in tumour relapse and metastasis. Like normal stem cells, CSCs possess long-term self-renewal and multi-lineage differentiation potential. To prevent relapse and metastasis, it is critical to identify molecular targets that regulate CSC maintenance and self-renewal.

Post-translational modification of proteins by the small ubiquitin-like modifier (SUMO) family is frequently dysregulated in cancer and is required for tumour growth and metastasis^10^,^11^. SUMOylation involves several steps that are catalysed by three enzymes: SUMO activating enzyme (E1, a heterodimer of SAE1 and SAE2 (also known as Uba2) subunits); SUMO conjugating enzyme (E2, also known as Ubc9 or UBE2I); and 1 of ∼10 E3 ligases^12^. Briefly, a SUMO protein is first activated by its E1 through ATP hydrolysis, and then forms a thioester conjugate with the E1. SUMO is then transferred to E2, forming a thioester conjugate with E2. Finally, SUMO is transferred to a target protein, a step usually stimulated by an E3 ligase. Ultimately, SUMO modification adds a new docking site to target proteins, and thus enables new protein–protein interactions through the SUMO-interacting motif during signalling events^13^,^14^. SUMOylation enzymes are present at higher levels in cancer cells than in normal cells; these high levels are required for tumour progression and metastasis, and are associated with poor survival^15^,^16^. However, the role of SUMOylation in CSC maintenance and self-renewal is poorly understood.

In this study, we investigated the role of the SUMO E1 in regulating CSC maintenance and self-renewal. Aldehyde dehydrogenase (ALDH) activity is a widely occurring CSC marker in different cancer types, including solid tumours (for example, colon, lung, liver, bone, pancreatic, prostate, head and neck, bladder, thyroid, brain, melanoma and cervical tumours) and haematological malignancies (for example, acute myeloid leukaemia)^17^,^18^,^19^,^20^,^21^,^22^,^23^,^24^,^25^,^26^,^27^,^28^. ALDH activity also plays an important role in CSC biology^29^. We discovered that SUMO E1 and global SUMOylation levels were much higher in CSCs than in non-CSCs of colorectal cancer (CC) cells. Knockdown of SAE2, the catalytic subunit of the SUMO E1, in CSCs reduced their tumour initiation capability *in vitro* and in xenograft models. Mechanistic investigations revealed that expression of ALDH1A1, an isoform believed to be critical for CSC function in many cancer types^30^, was reduced by knockdown of SAE2. We further found that degradation of octamer-binding transcription factor 1 (Oct-1, encoded by POU2F1), the transcriptional activator of ALDH1A1 (refs 31, 32), was increased by SAE2 knockdown. This was not through direct Oct-1 SUMOylation; rather, we identified tripartite motif-containing protein 21 (TRIM21) as the ubiquitin E3 ligase for Oct-1. Expression of TRIM21 was increased on knockdown of SAE2, leading to increased Oct-1 ubiquitination and degradation. We verified that TRIM21 expression is dependent on the transcription factor interferon regulatory factor 1 (IRF1), which is regulated by SUMOylation^33^,^34^. Therefore, the regulation of Oct-1 stability by SUMOylation is through SUMO-dependent expression of the ubiquitin E3 ligase (that is, TRIM21) that enhances Oct-1 ubiquitin-dependent proteasome degradation. Taken together, we have identified a novel SUMO-dependent mechanism for protein stability control and CSC maintenance. Our findings suggest that SUMOylation, in particular the SUMO E1, may be an effective therapeutic target for inhibiting CSC maintenance and self-renewal.

## Results

### Clinical samples indicates a key role of SUMOylation in CC CSCs

To define which SUMOylation-related proteins have altered expression in CC, we first examined mRNA levels in CC cell lines in comparison with normal colonic mucosa (Supplementary Table 1). We used HCT116 and HT29 lines, which are representative of major CC types (for example, they have microsatellite instability or are DNA mismatch repair deficient). All of the SUMO-related proteins investigated (SUMO-1, -2 and -3; both SUMO E1 subunits (SAE1 and SAE2/UBA2); Ubc9 (E2, UBE2I); the E3 ligases PIAS1, PIAS2, PIAS3, PIAS4, RanBP2 and MMS21; and de-SUMOylation enzymes SENP1, SENP2, SENP3, SENP5, SENP6 and SENP7) demonstrated increased expression in CC cell lines relative to normal colonic mucosa. The two SUMO E1 subunits, SAE1 and SAE2, were the most highly elevated. To confirm this finding, we examined 27 published gene expression data sets of CC primary tissues; increased SAE2 expression was observed in the majority of studies (Supplementary Fig. 1). To examine the protein levels of the SUMOylation-related proteins, we carried out immunohistochemistry (IHC) on stage II and III colorectal tumour specimens (*n*=51) and matched normal tissues (IRB 10132 with patient consent). Our analyses confirmed that SAE2 and SAE1 were more elevated in malignant as compared with normal tissues than the SUMO E3 PIAS1 (Supplementary Fig. 2).

To understand the link between expression of SUMO E1 and CC resistance to chemoradiation, we assessed archived tissues from rectal cancer patients before and after neoadjuvant chemoradiation therapy in a Phase II trial (*n*=18) for SUMOylation-related proteins using IHC. Semiquantitative ‘quickscores' (QS=staining area multiplied by intensity, with values of 0–18) were calculated for IHC specimens. When comparing pre- and post-neoadjuvant chemoradiation tissues from the same patient, there was a significant increase in SAE1 and SAE2 but not PIAS1 (Table 1). This indicates that increased expression of SUMO E1 is associated with CC cells that are resistant to chemoradiation. Because chemoradiation increases the CSC population^7^,^8^,^9^, higher SUMO E1 levels after chemoradiation suggests its higher levels in CSCs.

### The SUMO E1 is required for CSC maintenance and self-renewal

**Figure 1:**
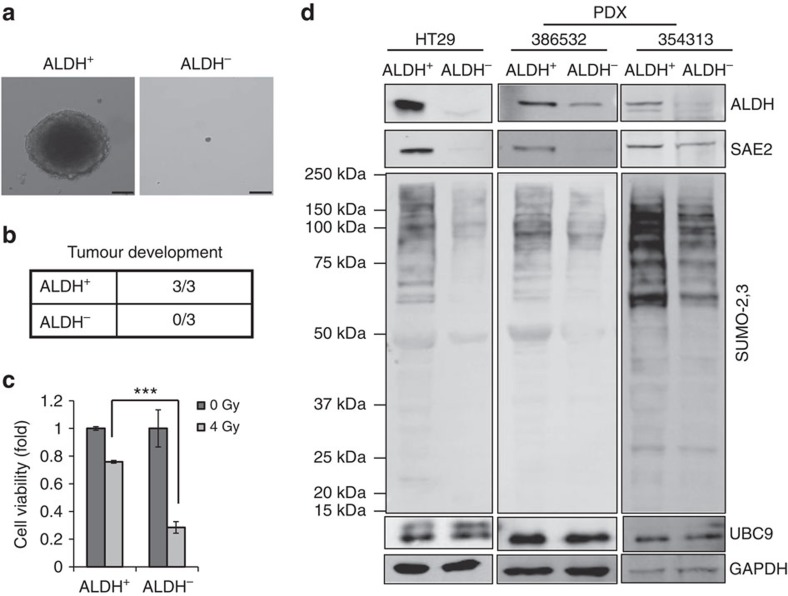
Colorectal cancer stem cells have higher SAE2 and global SUMOylation levels than non-cancer stem cells. (**a**) Representative colony formation assay of ALDH^+^ and ALDH^−^ cells isolated from HT29 cells (scale bar, 50 μm). (**b**) Tumour development of ALDH^+^ and ALDH^−^ cells isolated from HT29 that were injected in NSG mice (500 cells per mice) subcutaneously. (**c**) Cell viability assay of ALDH^+^ and ALDH^−^ cells after irradiation (4 Gy) (***P<0.001). (**d**) Western blot showing ALDH, SAE2, Ubc9 and global SUMOylation (SUMO-2,3) level of ALDH^+^ and ALDH^−^ cells isolated from colorectal cancer cell line HT29 and primary colon cancer PDX tumour tissue (386532 and 354313 correspond to PDX tissues from different patient specimen); GAPDH, loading control.

To analyse SUMOylation in CC CSCs, we first validated published markers for isolating CSCs. CSC markers reported for CC include CD133, CD44, LGR5 and ALDH activity^3^,^22^,^35^,^36^. Previous studies also validated HT29 as containing significant amount of CSCs^35^. ALDH^+^ and ALDH^−^ HT29 cells were sorted and tested for colony formation in Matrigel-based three-dimensional culture. ALDH^+^ cells formed colonies but ALDH^−^ cells failed to grow colonies (Fig. 1a and Supplementary Fig. 3). We determined tumour-initiating ability of ALDH^+^ and ALDH^−^ HT29 cells using *in vivo* mouse models. Injection of ALDH^+^ cells caused tumour growth in all NSG mice tested (3/3), but injection of ALDH^−^ cells failed to grow tumours (0/3) (Fig. 1b). Consistent with CSC characteristics, ALDH^+^ cells were more resistant to radiation than were ALDH^−^ cells (Fig. 1c).

Western blot analysis for ALDH confirmed successful sorting of HT29 and patient-derived xenograft (PDX) primary cells (Fig. 1d). HT29 CSCs (ALDH^+^) expressed greater levels of SAE2 and global SUMOylation than non-CSCs (ALDH^−^) (Fig. 1d and Supplementary Fig. 3d). Interestingly, differences in levels of other SUMOylation-related enzymes, including Ubc9 (Fig. 1d) and PIAS1 (Supplementary Fig. 3b), were less pronounced than differences in SAE2 levels between HT29 CSCs and non-CSCs. However, SENP2 levels are higher in ALDH^+^ than in ALDH^−^ cells, suggesting that ALDH^+^ cells also have enhanced dynamics of SUMO conjugation and deconjugation. To confirm that the results were cell line-independent, we performed the same experiment in primary cultures of PDX CC cells. In PDX primary cells, higher SAE2 and SUMOylation levels were observed in ALDH^+^ than in ALDH^−^ cells (Fig. 1d (SAE2, SUMO-2/3) and Supplementary Fig. 3d (SUMO-1)).

**Figure 2:**
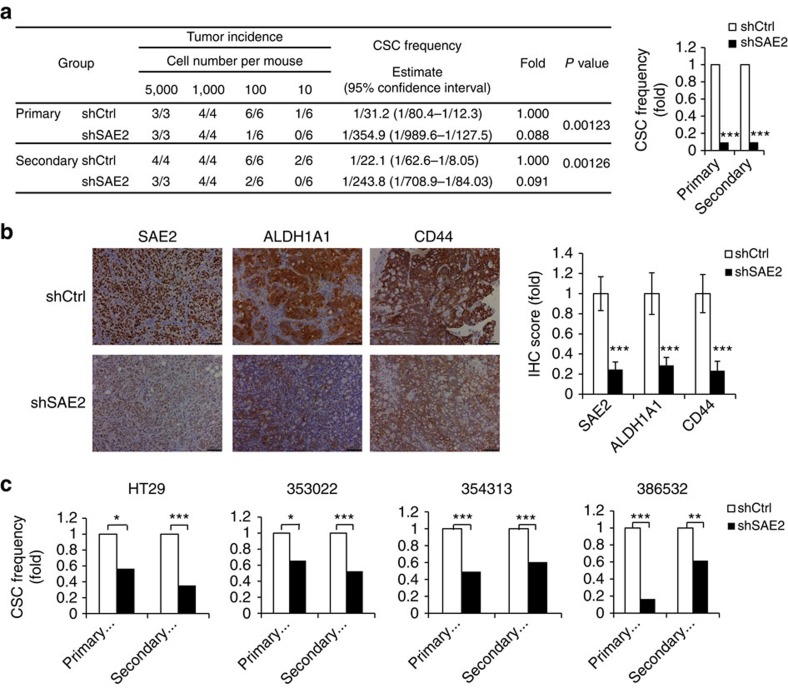
SAE2 knockdown impairs maintenance and self-renewal of CSCs. (**a**) Frequency of CSC in HT29 cells transduced with control non-silencing shRNA (shCtrl) or shSAE2 lentivirus measured by LDA *in vivo*. Left panel shows detailed data in tables and right panel shows a plot of CSC frequency. (**b**) IHC staining of SAE2, ALDH1A1 and CD44 of tumour in shCtrl and shSAE2 group (xenografted with 1,000 cells per mouse, scale bar, 100 μm). Quantification of IHC staining is shown to the right. (**c**) Frequency of spheroid-initiating cells measured by LDA in HT29 and primary culture of CC PDX (353022, 354313 and 386532) with SAE2 knockdown (shSAE2). Fold was in comparison to the CSC frequency of shCtrl group. *P<0.05, **P<0.01 and ***P<0.001.

The enrichment of SAE2 in CC CSCs suggests that the SUMO E1 plays an important role in CSC maintenance and self-renewal. To confirm the importance of SAE2 in CSC maintenance, we performed *in vivo* limited dilution assays (LDAs) to monitor tumour initiation as described^36^,^37^. HT29 cells stably expressing SAE2-targeting shRNA or control shRNA were injected into NSG mice in a limited dilution series, and tumour incidence was monitored over 2 months. SAE2 knockdown led to more than 90% reduction of CSC frequency *in vivo* (Fig. 2a). To determine the effect of SAE2 knockdown on self-renewal of CSCs, we carried out secondary LDA in NSG mice by re-injecting cells dissected from the primary tumour as a limited dilution series and monitoring tumour development over 2 months. The secondary LDA showed that SAE2 knockdown resulted in more than 90% reduction in self-renewal frequency of CSC (Fig. 2a) and reduced tumour weight (Supplementary Fig. 4a). Consistent with SAE2 knockdown, IHC staining of tumours in the shSAE2 group showed reduced level of SAE2, as well as reduced level of the CSC markers ALDH1A1 (an isoform believed to be critical for ALDH activity in CSCs^30^) and CD44, relative to shCtrl cells (Fig. 2b). HT29 shCtrl and shSAE2 cells were used in spheroid initiation assay as an *in vitro* LDA for comparison with *in vivo* LDA. Similar to *in vivo* LDA, HT29 SAE2 knockdown also decreased CSC frequency as determined by *in vitro* LDA, a validation of the *in vitro* LDA method (Fig. 2c, left panel). Similarly, on SAE2 knockdown, three different PDX primary cultures showed 50–80% reduction in CSC frequency in primary and secondary *in vitro* LDAs (Fig. 2c and Supplementary Fig. 4b,c). These results indicate that the importance of the SUMO E1 in CC CSCs is cell line-independent.

**Figure 3:**
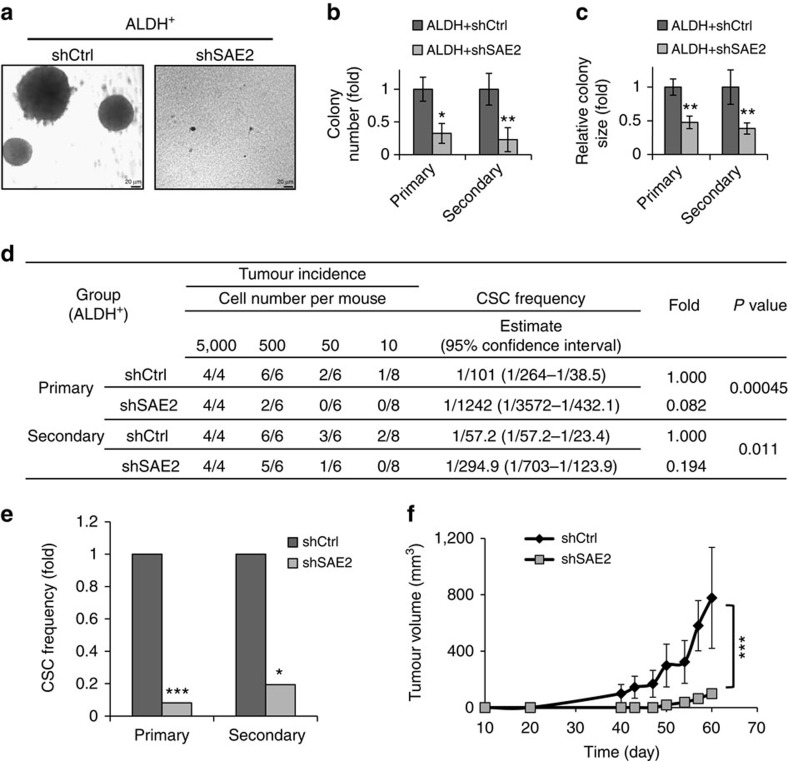
SAE2 knockdown impairs self-renewal and cancer initiation of CSC. (**a**) Representative colony formation assay of HT29 ALDH^+^ cells transduced with control non-silencing shRNA (shCtrl) or shSAE2 lentivirus and grown in Matrigel (scale bar, 20 μm). (**b**,**c**) Quantification of colony number (**b**) and colony size (**c**) from four different wells and six fields in each well of the experiments described in **a**. (**d**,**e**) Frequency of CSC in HT29 ALDH^+^ cells transduced with shCtrl or shSAE2 lentivirus measured by LDA *in vivo* as shown by the detailed data (**d**) and as a plot of fold changes in the CSC frequency (**e**). (**f**) Tumour growth curve for HT29 ALDH^+^ shCtrl or shSAE2 cells injected subcutaneously with 500 cells per mouse of six mice in each group. *P<0.05, **P<0.01 and ***P<0.001.

To investigate the role of SAE2 in CSCs directly, we performed knockdown of SAE2 in ALDH^+^ cells and carried out LDA *in vitro* and *in vivo*. SAE2 knockdown caused reduced colony formation and smaller colony sizes in three-dimensional Matrigel culture, indicating significant impairment of tumour-initiating ability in CSCs. To investigate self-renewal of CSCs, cells from the primary colony were propagated in a secondary colony formation assay (Fig. 3a, right panel). Again, SAE2 knockdown reduced colony number and colony size in the secondary colony formation assay, indicating that SAE2 knockdown significantly impaired self-renewal of CSCs (Fig. 3b,c). In addition, transduction of SAE2 shRNA lentivirus into ALDH^+^ HT29 cells suppressed both tumour initiation and growth in *in vivo* LDA (Fig. 3d,e). Furthermore, tumour growth was significantly suppressed in ALDH^+^ cells on knockdown of SAE2 (Fig. 3f). IHC staining of tumour tissues confirmed knockdown of SAE2 level and reduced levels of CSC markers (ALDH1A1 and CD44) (Supplementary Fig. 5).

### SUMOylation regulates ALDH through Oct-1

**Figure 4:**
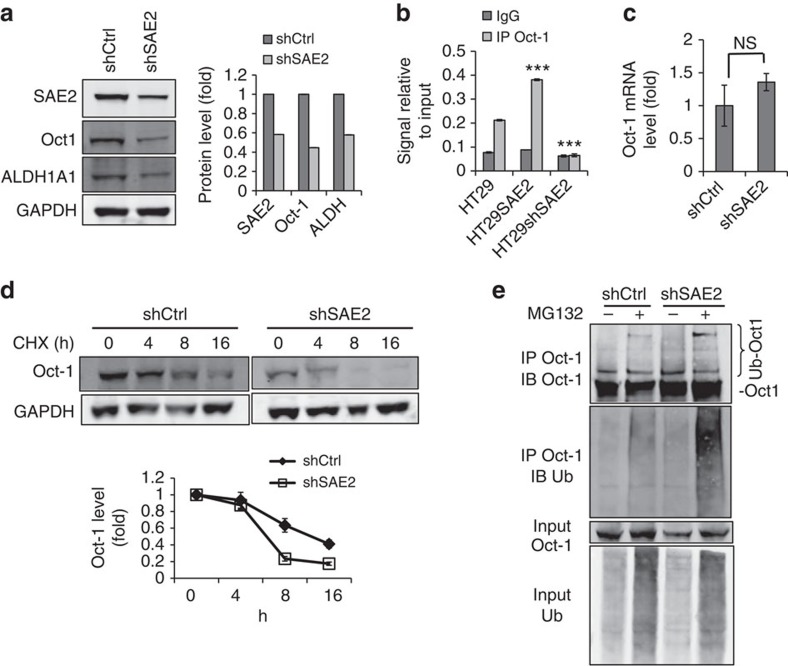
SAE regulates ALDH expression through controlling Oct-1 stability. (**a**) Representative western blot of stable HT29 line transduced with shSAE2 lentivirus showed lower SAE2, Oct-1 and ALDH1A1 protein levels than HT29 transduced with control non-silencing shRNA (shCtrl); GAPDH, loading control. Right panel: quantification of band intensity. (**b**) SAE2 knockdown decreased and SAE2 overexpression increased the occupancy of Oct-1 on the ALDH1A1 promoter as measured by ChIP assay. (**c**) SAE2 knockdown did not affect Oct-1 transcription as shown by real-time quantitative PCR measurement of mRNA of HT29 shCtrl and shSAE2 cells. (**d**) Representative western blot showing SAE2 knockdown reduced Oct-1 stability, as shown by treatment with 100 μg ml^−1^ CHX to block protein synthesis in HT29 shCtrl and shSAE2 cells; GAPDH, loading control. Quantification of band intensities (Oct-1 decay curve) in three independent experiments is shown below. (**e**) Representative western blot showing SAE2 knockdown-enhanced Oct-1 ubiquitination. HT29 shCtrl or shSAE2 cells were treated with or without proteasome inhibitor MG132 (10 μM) for 8 h, and IP was carried out using cell lysates, followed by western blot with an anti-Oct-1 or anti-ubiquitin antibody. NS, not significant; ***P<0.001.

Next we investigated how knockdown of SAE2 reduced ALDH1A1 protein level. We found that knockdown of SAE2 in HT29 cells reduced the protein level of Oct-1, a transcriptional activator of ALDH1A1 and several other ALDH isoforms^31^,^32^ (Fig. 4a). Chromosome immunoprecipitation (ChIP) assay showed that the occupancy of Oct-1 at the ALDH1A1 promoter increased by SAE2 overexpression (HT29SAE2) and was reduced by SAE2 knockdown (HT29 shSAE2) (Fig. 4b). These results suggest that SUMOylation regulates ALDH1A1 expression through Oct-1. Knockdown of SAE2 did not reduce Oct-1 mRNA level, indicating that SUMOylation does not directly regulate Oct-1 gene expression (Fig. 4c). However, knockdown of SAE2 increased Oct-1 protein degradation (Fig. 4d). In addition, SAE2 knockdown increased Oct-1 ubiquitination (Fig. 4e). These results indicate that SAE2 knockdown led to increased Oct-1 ubiquitination-dependent degradation. SUMOylation-dependent Oct-1 degradation is unlikely due to SUMOylation of Oct-1 itself, as we could not observe Oct-1 SUMOylation (Supplementary Fig. 6a).

### Identification of TRIM21 as Oct-1 ubiquitin E3 ligase

**Figure 5:**
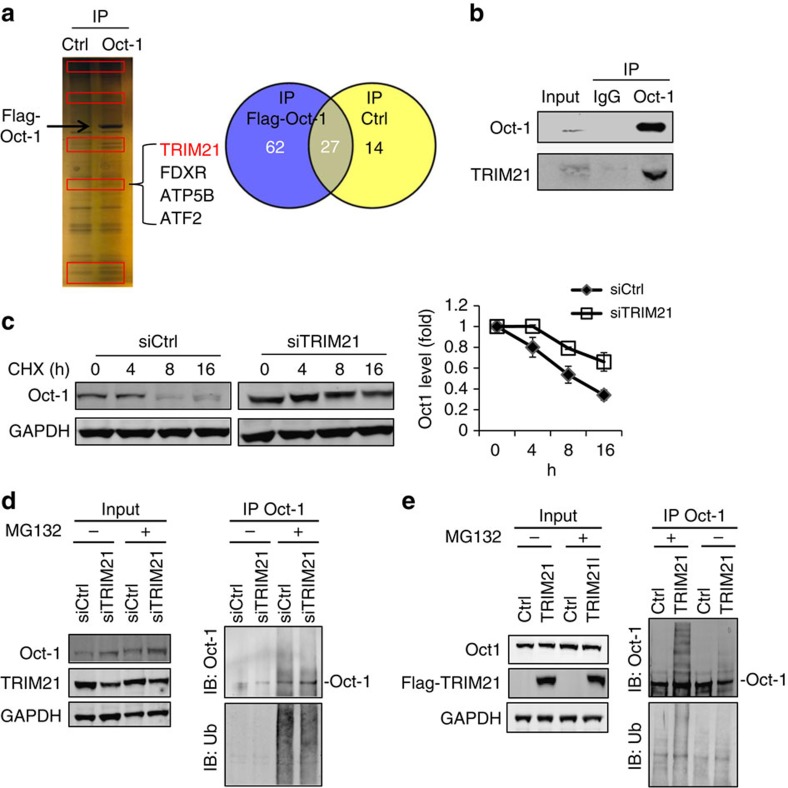
Identification of TRIM21 as an Oct-1 ubiquitin E3 ligase. (**a**) Mass spectrometry identified TRIM21 as an Oct-1-interacting protein. Representative silver stain of HCT116 cells transfected with Flag-tagged Oct-1 or empty vector (Ctrl) for 2 days, lysed and immunoprecipitated with anti-Flag antibody-conjugated M2 beads. Proteins identified in each group are indicated in the graphic. (**b**) Representative IP-western blot analysis of the interaction between endogenous TRIM21 and Oct-1 in HT29 cells. (**c**) Knockdown of TRIM21 delayed Oct-1 degradation. Representative western blot of Oct-1 level over time in HT29 cells transfected with control non-silencing siRNA (siCtrl) or siTRIM21 for 3 days, followed by 100 μg ml^−1^ CHX treatment; GAPDH, loading control. Oct-1 decay curve (right panel) was determined by quantifying three independent experiments. (**d**) TRIM21 knockdown resulted in increased Oct-1 protein level and decreased ubiquitination of Oct-1. Representative western blot of HT29 cells transfected with siCtrl and siTRIM21 treated with or without 10 μM MG132 for 8 h. IP was carried out with cell lysates, and Oct-1, TRIM21 and ubiquitin were detected; GAPDH, loading control. (**e**) Overexpression of TRIM21 decreased Oct-1 and increased Oct-1 ubiquitination in cells. Representative western blot of HT29 cells were transfected with empty vector (Ctrl) or Flag-TRIM21 for 2 days, then treated with or without 10 μM MG132 for 8 h. IP was carried out with cell lysates, and western blot was performed to detect Oct-1, TRIM21 and ubiquitin; GAPDH, loading control.

The mechanism of Oct-1 degradation has not been previously reported. To identify the ubiquitin E3 ligase targeting Oct-1 degradation, HCT116 cells were transfected with Flag-tagged Oct-1 (Oct-1) or empty vector (Ctrl) for 2 days. For this experiment, we used HCT-116 cells, which have higher transfection efficiency than HT29 for producing Flag-tagged Oct-1. Cell lysates were used for immunoprecipitation (IP) with an anti-Flag-tag antibody. After washing, Oct-1 was eluted with a Flag-tag peptide, followed by tryptic digestion and liquid chromatography-tandem mass spectrometry liquid chromatography-tandem mass spectrometry (LC–MS/MS) analysis. Many fewer proteins were pulled down in control cells than Flag-Oct-1 expressing cells, a validation of the method (Fig. 5a). In addition, Oct-1 was pulled down from Oct-1-expressing cells but not from control cells, validating the experimental approach (Fig. 5a). Using mass spectrometry to identify the Oct-1-interacting proteins, we found only one ubiquitin E3 ligase, TRIM21 (Fig. 5a and Supplementary Table 2)^38^. The interaction between endogenous Oct-1 and TRIM21 was verified in HT29 cells by co-IP with an anti-Oct-1 antibody (Fig. 5b). To directly test the effect of TRIM21 on endogenous Oct-1 protein stability, we knocked down TRIM21 in HT29 cells and observed an increase of Oct-1 protein stability (Fig. 5c). TRIM21 knockdown also reduced Oct-1 ubiquitination and increased free Oct-1 level (Fig. 5d). To further investigate whether TRIM21 functions as E3 ligase that enhances Oct-1 ubiquitination, we overexpressed TRIM21 in HT29 cells and treated the cells with MG132 to prevent protein degradation before performing an ubiquitination assay. A significant increase of polyubiquitinated Oct-1 protein was observed in TRIM21-transfected cells (Fig. 5e).

We did not observe significant stimulation of Oct-1 ubiquitination by TRIM21 in *in vitro* biochemical assays using purified proteins (Supplementary Fig. 6b,c), possibly due to a requirement for proteins in addition to TRIM21 for ubiquitination of Oct-1, or due to a lack of activity in recombinant TRIM21.

**Figure 6:**
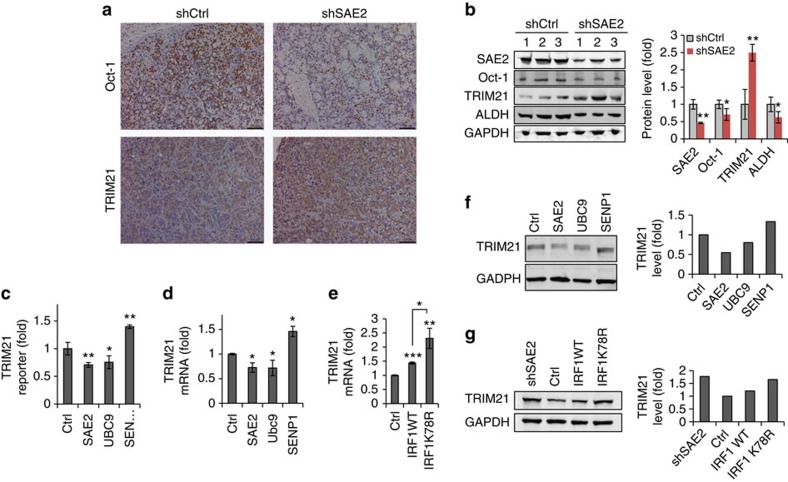
SUMOylation regulates TRIM21 expression through IRF1. (**a**) Representative IHC staining indicates higher TRIM21 level corresponding with lower Oct-1 in shSAE2 group tumour tissue compared with shCtrl group (scale bar, 100 μm). Tumour tissues were from LDA assay described in Fig. 2b. (**b**) Western blot (left) and quantification of SAE2, Oct-1, TRIM21 and ALDH levels in the same tumour tissues as **a**; GAPDH, loading control. ‘1, 2 and 3' indicate tumour tissues from different mouse. (**c**) TRIM21 promoter activity was inhibited by overexpression of SAE2 and Ubc9 but increased by SENP1 overexpression as determined by luciferase reporter assay. HT29 cells were transfected with empty vector (Ctrl) or SAE2, UBC9 or SENP1 expression plasmid together with TRIM21 promoter luciferase reporter and *Renilla* plasmids. Dual-luciferase activity was measured after 48 h and normalized results were analysed with two-tailed Student's *t*-test. (**d**) TRIM21 mRNA level was suppressed by SAE2 or Ubc9 overexpression and enhanced with SENP1 overexpression in HT29 cells as determined by quantitative PCR (qPCR). (**e**) IRF1 SUMOylation site mutant K78R induced higher TRIM21 mRNA level than wild-type (WT) IRF1 as determined by qPCR. (**f**) TRIM21 protein level was suppressed on SAE2 or Ubc9 overexpression but enhanced with SENP1 overexpression as indicated by western blot; GAPDH, loading control. (**g**) Western blot showed overexpression of K78R mutant-induced higher TRIM21 protein level than WT IRF1 in shCtrl HT29 cells; GAPDH, loading control. *P<0.05, **P<0.01 and ***P<0.001.

The correlation between Oct-1 and TRIM21 levels was also observed in xenograft experiments. Knockdown of SAE2 reduced Oct-1 and increased TRIM21 protein levels in mouse xenograft tumour tissues as shown by IHC staining (Fig. 6a). This was confirmed by western blots for Oct-1, TRIM21, ALDH and SAE2 in the mice tumour tissues from *in vivo* LDA (Fig. 6b). Overexpression of SAE2 or Ubc9 decreased TRIM21 promoter activity, while overexpression of SENP1 increased TRIM21 promoter activity, suggesting that SUMOylation suppressed TRIM21 gene expression (Fig. 6c). Consistent with this, TRIM21 mRNA level was suppressed on SAE2 or Ubc9 overexpression but enhanced with SENP1 expression in HT29 cells (Fig. 6d). IRF1, a transcriptional activator of TRIM21 (ref. 33), is SUMOylated at K78 (ref. 39), and previous reports show that its transcription activity is suppressed by SUMOylation^34^. Consistent with the previous finding, the SUMOylated IRF1 band could be observed in ALDH^+^ and not in ALDH^−^ cells (Supplementary Fig. 7a). A SUMOylation-defective IRF1 mutant K78R induces higher TRIM21 mRNA levels than does WT IRF1, suggesting that SUMOylation suppresses TRIM21 expression (Fig. 6e). Consistent with this, TRIM21 protein levels were suppressed on SAE2 or Ubc9 overexpression but enhanced with SENP1 overexpression in cells (Fig. 6f). In addition, the IRF1-K78R mutant induced higher TRIM21 protein expression than wild-type IRF1 (Fig. 6g). TRIM21 and Oct-1 levels are reversely correlated in both HT29 cell lines and PDX primary cells (Supplementary Fig. 7b). Taken together, our data indicate that inhibiting SUMOylation can increase IRF1 transcriptional activity, which results in increased TRIM21 expression and enhanced ubiquitination and degradation of Oct-1.

**Figure 7:**
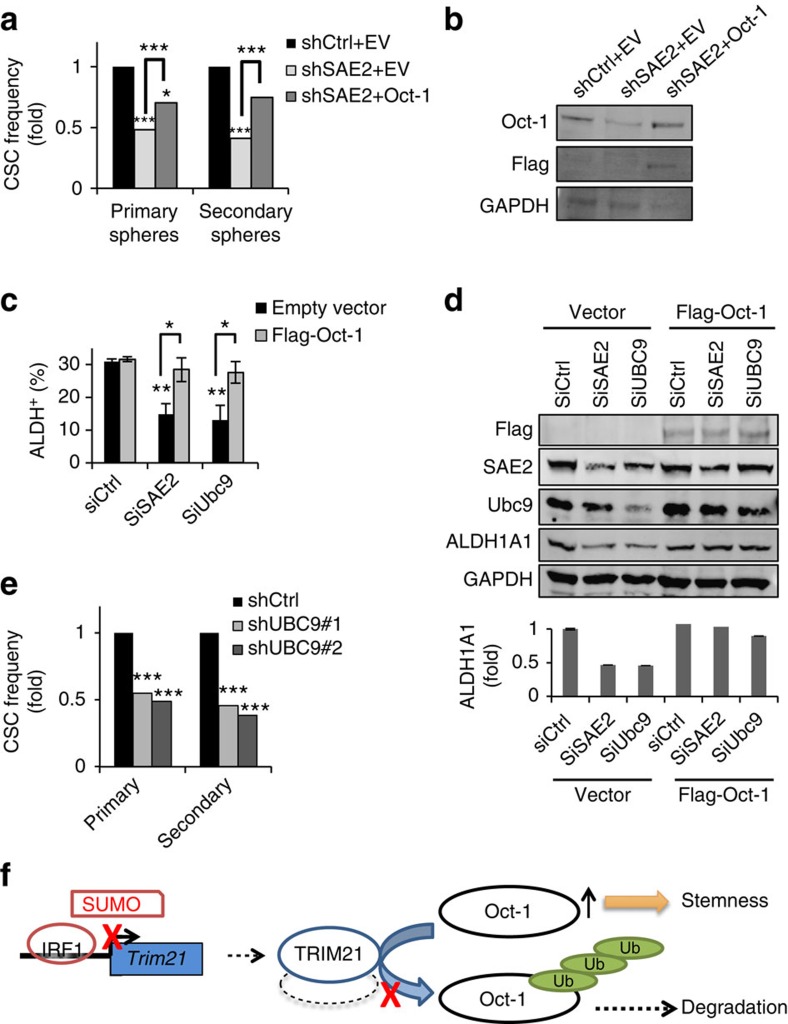
A mechanism of how SUMOylation is involved in CSC function. (**a**) Overexpression of Oct-1 partially compensated for the reduction of CSC frequency by SAE2 knockdown in HT29 cells as determined by LDA. Stable cell lines were generated with lentivirus expressing pLenti CMV-hygro empty vector (EV) or pLenti CMV-Flag-Oct-1 in HT29 shCtrl and shSAE2 cells as shCtrl+EV, shSAE2+EV and shSAE2+Oct-1. (**b**) Representative western blot of the stable lines to confirm expression with Oct-1 and Flag-tag antibodies; GAPDH, loading control. (**c**) Overexpression of Oct-1 in SAE2 or Ubc9 knockdown cells restored ALDH^+^ cells population in HT29 cells as measured by FACS analysis using the AldeFluor kit. HT29 cells were transfected with control non-targeting siRNA (SiCtrl), SAE2-targeting siRNA (SiSAE2) or Ubc9-targeting siRNA (SiUbc9) followed by Flag-Oct-1 plasmid or control empty vector transfection. After 3 days, cells were collected for FACS analysis using the AldeFluor kit. (**d**) Representative western blot of the samples from **c**; quantification of the ALDH1A1 band intensity is shown on the bottom. (**e**) Knockdown of Ubc9 reduced CSC frequency, as shown by LDA using spheroid formation using HT29 stable cell lines expressing two different UBC9-targeting shRNA (shUBC9#1 and shUBC9#2). (**f**) Schematic diagram showing the mechanism of how SUMOylation regulates CSCs through Oct-1, TRIM21 and IRF1. *P<0.05, **P<0.01 and ***P<0.001.

To further establish the connection between SAE2 and ALDH1A1 through Oct-1, we investigated whether overexpression of Oct-1 in SAE2 knockdown cells could restore ALDH activity and CSC population and self-renewal. We generated stable cell lines expressing an empty vector or Flag-tagged Oct-1 in HT29 shCtrl and shSAE2 cells (Figs 2 and 7a). The stable lines were confirmed by western blot (Fig. 7b). Overexpression of Oct-1 partially compensated for the effect of SAE2 knockdown in CSC maintenance and self-renewal in *in vitro* LDA (Fig. 7a and Supplementary Fig. 8). In addition, overexpression of Oct-1 in SAE2 or Ubc9 knockdown cells restored the population of ALDH^+^ cells (Fig. 7c) and ALDH1A1 protein levels (Fig. 7d) in HT29. The important role of SUMO E1 in CSC maintenance and self-renewal is likely due to SUMOylation-dependent functions of SAE2, because knockdown of Ubc9 by two independent shRNAs also reduced CSC maintenance and self-renewal, as shown by *in vitro* LDA (Fig. 7e and Supplementary Fig. 9a). Knockdown of SAE2 or Ubc9 also inhibited cell proliferation (Supplementary Fig. 9b). The regulation of self-renewal is not necessarily related to cell proliferation. LDA *in vitro* and *in vivo* was previously shown to measure stemness of CSC independent of proliferation^36^. In addition, knockdown of SUMO-1, -2 or -3 also reduced ALDH^+^ cell population and ALDH1A1 protein level (Supplementary Fig. 10).

## Discussion

In this study, we have shown that SUMOylation is critical to CSC maintenance and self-renewal. We have demonstrated that SUMOylation regulates ALDH1A1 expression, a CSC marker that is important for CSC maintenance and self-renewal^29^,^30^, through regulating the IRF1-dependent expression of TRIM21 (Fig. 7f). We identified TRIM21 as an ubiquitin E3 ligase that controls the degradation of Oct-1, which is a transcription factor for the expression of ALDH1A1. The important role of SUMOylation in CSC as shown in this study is consistent with a previous study suggesting a positive correlation between global SUMOylation and expression of Lin28, a protein that is highly expressed in stem cells as well as in cancer cells^40^. In addition, SUMOylation is important for normal colon stem cell self-renewal^41^, and pathways controlling CSC are often similar to those controlling normal stem cells. The clinical significance of this finding is suggested by the analysis of clinical samples and PDX primary cells (Fig. 1, Table 1 and Supplementary Figs 1–3). Importantly, SUMO E1 level increased after chemoradiation in patient primary CC tumour tissues; such an increase is likely associated with an increase in CSC population and chemoradiation resistance.

Because ALDH activity has been found as a marker for CSCs in many cancer types^17^,^18^,^19^,^20^,^21^,^22^,^23^,^24^,^25^,^26^,^27^,^28^, it is likely that our findings are applicable to a broad range of cancers. To investigate this possibility, we isolated ALDH^+^ and ALDH^−^ cells from the breast cancer cell line HCC1937, and found that ALDH^+^ cells had higher levels of SAE2 and global SUMOylon than ALDH^−^ cells. In addition, SAE2 knockdown in HCC1937 cells decreased the population of ALDH^+^ cells (Supplementary Fig. 11).

In this study, we also uncovered a unique mechanism of SUMOylation-dependent regulation of protein stability. SUMOylation regulates Oct-1 stability, not through direct modification of Oct-1, but through altering the expression of its ubiquitin E3 ligase, TRIM21. This SUMOylation-dependent control of a ubiquitin E3 ligase is distinct from the well-established paradigm of SUMO-dependent ubiquitin E3 ligase-induced ubiquitination and proteasome degradation; for example, RNF4, a prototype of such ubiquitin E3 ligases, targets SUMOylated proteins for ubiquitination and proteasome degradation through recognition of poly-SUMO chains^42^,^43^,^44^,^45^. Our results revealed a unique mechanism of SUMO-dependent ubiquitination and degradation.

The findings described here expand on previous finding that SUMOylation, and the SUMO E1 in particular, is potentially an important target for developing anticancer therapies^10^,^11^,^46^,^47^,^48^. SUMOylation likely affects multiple targets that are important for CSC maintenance and self-renewal. Indeed, we showed that knockdown of SAE2 reduced levels of another colorectal CSC marker, CD44 (Fig. 2b and Supplementary Fig. 5). This likely occurs through a different mechanism than that described here, as CD44 is a target gene of the Wnt-signalling pathway^49^. Previous studies identified key factors in the Wnt-signalling pathway as substrates of SUMO modification^50^,^51^,^52^,^53^. Further studies on regulation of Wnt pathway by SUMOylation are required to clarify this interaction. Recent studies also revealed that SUMOylation, in particular the SUMO E1, has a critical role in promoting KRas- and Myc-driven tumorigenesis^10^,^54^. Although c-Myc activation contributes to up to 70% of all human cancers, and KRas mutation occurs in more than 50% of all human cancers, drugs inhibiting these oncogenes are not yet available. Our findings suggest that cancer therapeutics targeting SUMOylation could not only inhibit these major oncogenic drivers but also limit CSC growth and self-renewal.

## Methods

### Patient specimen IHC analysis

Colorectal tumour and matched normal tissues specimens from stage II and III colorectal cancer patients (*n*=51) were obtained from biopsy. After chemo and radiation therapy, tumour specimens were obtained from patients without complete pathologic response (non-pCR) (*n*=18). Archived specimens from patients with colorectal carcinoma (*n*=51), as well as archived normal colorectal tissue were subject to IHC staining using the following antibodies SAE2 (1:200, ab58451, Abcam), SAE1(1:100, ab56957, Abcam) and PIAS1(1:200, ab109388, Abcam). Omission of the primary antibody was set as a negative control. IHC staining was evaluated by two independent pathologists who were blinded to patients' clinical outcome. The QS was calculated for each slide based on intensity and percentage of staining area. The intensity of staining was scored semiquantitatively as negative (0), weak (1), intermediate (2) or strong (3). The percentage of staining area was scored as 0–4% (1), 5–19% (2), 20–39% (3), 40–59% (4), 60–79% (5) and 80–100% (6). Two independent pathologists calculated QS by multiplying the intensity score with percentage of staining area score and the average score was obtained for each slide. *P* values were derived using two-tailed Student's *t*-test and uncertainties were indicated as s.d. The study was approved by the Research Ethics Board at the City of Hope (IRB # 10132).

### Cell lines and PDX primary culture

Colorectal cancer cell lines HT29 and HCT116 (obtained from American Type Culture Collection) were grown in DMEM. Media were supplemented with 10% heat-inactivated fetal calf serum (Omega Scientific, Inc.), 2 mM L-glutamine, 100 U ml^−1^ penicillin and 100 μg ml^−1^ streptomycin. Cells were routinely tested by using Mycoalert mycoplasma detection kit (LT07-418, Lonza) to confirm the absence of mycoplasma species.

The PDX model was generated by a subcutaneous implant of human colorectal tumour tissues into NSG mice. The collection of human colorectal cancer tissue was approved by the Research Ethics Board at the City of Hope (IRB13389). Xenograft tumour tissue was washed in PBS, minced and incubated with collagenase (235 U ml^−1^) and hyaluronidase (850 U ml^−1^) (Sigma-Aldrich) for 90–120 min at 37 °C. DMEM with 10% FBS was added to stop enzymatic digestion. The sample was serially filtered through 70 and then 40 μm cell strainers. Cells were spun down and re-suspended with 1 × ice-cold red blood cell lysis buffer (Santa Cruz Tech) and incubated for 2 min to lyse red blood cells. Cells were then used for further studies. For further separation of cancer cells from stromal or fibroblasts, magnetic sorting was carried out using EpCAM positive selection kit (StemCell Tech).

### Aldefluor assay and fluorescence-activated cell sorting

The Aldefluor kit (StemCell Tech) was used to isolate cells with high ALDH activity using fluorescence-activated cell sorting. Briefly, cells were suspended in Aldefluor assay buffer containing BODIPY-aminoacetaldehyde and incubated (30 min, 37 °C). Control samples were incubated with buffer containing 15 μM diethylaminobenzaldehyde, an ALDH inhibitor. For cell lines HT29 and HCC1937, the Aldefluor assay was performed to sort ALDH^+^ and ALDH^−^ populations. For colorectal cancer cells isolated from PDXs, anti-EpCAM antibody (Alexa647-conjugated, #5447, Cell Signaling Technology, Inc.) was used to eliminate stromal or fibroblasts cells before the Aldefluor assay. Cell sorting was conducted with an ARIA III cell sorter and the results were analysed with Summit software.

### Tumorigenicity in NSG mice

For the generation of xenografts, cells were injected with Matrigel (BD Biosciences) subcutaneously into flanks of NSG mice (female, 6–8 weeks of age). Mice were monitored for 2 months to observe tumour formation and growth. Animal work was carried out in compliance with the ethical regulations approved by the Animal Regulation Committee, Beckman Research Institute, City of Hope, CA, USA (IACUC#10026).

### Matrigel colony formation assay

Single-cell suspensions were mixed 1:1 with Matrigel and plated in an eight-well chamber. After 2 weeks of incubation, colony formations were counted and measured using light microscopy (IX81 Olympus). Cells were isolated from colony in matrigel using Corning cell recovery solution (#354253, Corning) and re-seeded with matrigel for secondary colony formation. Colony number and size were counted and measured.

### Lentiviral vectors

A GIPZ shRNA-eGFP vector with a shRNA sequence targeting the 3′-untranslated region of SAE2 was purchased from GE Dharmacon (CloneId: V2LHS 68112). The GIPZ non-silencing lentiviral shRNA vector was used as a control (shCtrl). Both vectors have green fluorescent protein (GFP) and puromycin resistance markers. Two GIPZ shRNA-eGFP vectors were purchased from GE Dharmacon, one with shRNA sequence targeting the open reading frame of UBC9 (shUBC9#1, Clone ID V2LHS_171776), the other with shRNA sequence targeting the 3′-untranslated region of UBC9 (shUBC9#2, Clone ID V2LHS_171781). pLenti CMV/TO hygro empty (w214-1) vector was obtained from Addgene. Myc-DDK-tagged human Oct-1 (POU2F1) plasmid was purchased from Origene. DNA coding DDK-tagged Oct-1 was subcloned into pLenti CMV/TO hygro empty vector to make an Oct-1 expression plasmid (pLenti CMV-Oct-1), which was used for viral packaging of the Oct-1 expression construct with hygromycin resistance.

### DNA and RNA transfection

Transient transfection of plasmid DNA was performed using DNA transfection reagent (Lipofectamine LTX; Invitrogen). siRNA transfection was performed by using Lipofectamine RNAiMAX (Invitrogen). Cells were collected 48 h after plasmid DNA transfection and lysed directly in Laemmli sample buffer. After protein quantification, 0.7 M β-mercaptoethanol was added to the protein sample, which was boiled at 95 °C. For siRNA knockdown, cells were re-transfected with siRNA 72 h after the first transfection to ensure siRNA knockdown effects. The cells were then collected 72 h after siRNA transfection and either directly lysed in SDS buffer or the RNA was isolated with the microRNeasy kit (Qiagen) according to the manufacturer's instructions.

### Viral transduction of colorectal cancer cells

For lentiviral generation, the envelope plasmid pCMV-VSVG and the packaging plasmid pCMV-dR8.2-dvpr were obtained from Addgene (8454 and 8455, provided by Dr Bob Weinberg). 293T producer cells were transfected with the lentiviral expression vector and packaging DNAs by DNA transfection reagent (Lipofectamine LTX; Invitrogen). The supernatant containing lentiviral particles was collected 24–48 h after transfection.

For the HT29 cell line, lentiviral particles containing non-silencing or SAE2 shRNA were used for transduction and puromycin was added 48 h after transduction. Single colonies demonstrating strong GFP expression were picked for cell expansion. A stable line that constitutively expressed SAE2 shRNA (HT29 shSAE2) was established, and knockdown of SAE2 was confirmed by western blot. A stable control line (HT29 shCtrl) was also made with non-silencing shRNA containing lentivirus. Two stable lines in HT29 shUBC9#1 and shUBC9#2 were generated in the same way, and knockdown of UBC9 was confirmed by western blot. For the PDX primary cultured cancer cells, lentiviral particles that carried shSAE2 or Ctrl-shRNA expression vector were added and then cells were used for LDA 72 h after transduction. Both shSAE2 and Ctrl-shRNA vectors expressed GFP, which allowed tracking of transduced cells for each primary culture.

Stable cell lines were generated with lentivirus expressing pLenti CMV/TO hygro empty vector or pLenti CMV-Oct-1 in HT29 shSAE2 and shCtrl cells. The stable cell line HT29 shSAE2+Oct-1 was generated with simultaneous knockdown of SAE2 and overexpression of Oct-1. Stable control lines were also generated with transduction of pLenti CMV/TO hygro empty vector (EV) virus, and are referred to as HT29 shCtrl+EV and HT29 shSAE2+EV.

### Limited dilution analysis

For LDA *in vivo*, HT29 cell lines Ctrl-shCtrl and shSAE2 were dissociated into a single-cell suspension and diluted serially to the desired cell doses. Cells were injected subcutaneously into the flanks of NSG mice. Xenografted mice were monitored to up to 2 months to observe tumour formation and growth. Primary tumours from both shCtrl and shSAE2 groups were dissociated into single cells, and EpCAM magnetic sorting was used to isolate cancer cells from stromal cells and fibroblasts. Fluorescence-activated cell sorting analysis confirmed that more than 95% of the isolated cells were EpCAM^+^ and GFP^+^. The isolated cells were serially diluted to the desired cell doses and subcutaneously injected into NSG mice for secondary tumour growth. The number of tumours formed out of the number of mice injected was scored to determine the frequency of CSCs, which were calculated using the ELDA software provided by the Walter and Eliza Hall Institute (Melbourne, Australia). The same LDA *in vivo* study was performed with sorted ALDH^+^ cells. Briefly, ALDH^+^ cells from HT29 were transduced with shCtrl (control shRNA) or shSAE2 (SAE2 shRNA) lentivirus for 3 days. Then cells were injected to NSG mice in a limited dilution series and tumour incidence was monitored for 2 months. CSC frequency was determined and tumour growth curve was measured.

For *in vitro* LDAs, a single-cell suspension was made and serially diluted to different doses. For each dose, at least 24 wells were seeded with cells. For lower doses, 96 wells were plated at each dose. Four to six weeks later, wells containing spheres were counted, and the number of positive wells was used to calculate sphere-formation frequency using the ELDA software. The primary spheres were dissociated to single cells, serially diluted and seeded as above for secondary sphere formation and CSC frequency measurements.

### Chromatin immunoprecipitation (ChIP) assay

To detect the occupancy of Oct-1 at the human ALDH1A1 promoter, ChIP analysis was conducted. In all, 2 × 10^7^ cells were incubated in culture medium containing 1% formaldehyde (10 min, room temperature) and the crosslinking reaction was quenched with addition of glycine to a final concentration of 0.125 M. Cells were washed with PBS and collected, followed by sonication to obtain chromatin of primarily mononucleosome size. Fragmented chromatin was then incubated with anti-Oct-1 antibody at 4 °C overnight. Protein–DNA complexes were recovered using protein G agarose beads, and washed and eluted with elution buffer. Crosslinks were reversed in 0.25 M NaCl (overnight, 65 °C), and DNA was digested with proteinase K (2 h, 50 °C). The immunoprecipitated DNAs were subsequently isolated and used for PCR. PCR primers specific for the ALDH1A1 promoter were as follows: sense, 5′-GCTTCCTGCCCTAGGTGTTA-3′; antisense, 5′-GAACACAGGTGACTGGCTCA-3′.

### Western blot

Cells were lysed with Laemmli sample buffer (5% SDS, 25% glycerol, 150 mM Tris-HCl (pH 6.8) and 0.01% bromophenol blue). After protein concentration was measured by BCA protein assay, 0.7 M β-mercaptoethanol was added and protein samples were boiled for 10 min. Protein samples were separated by SDS–PAGE, and protein was transferred onto a polyvinylidene fluoride membrane (Immobilon-P membrane, Millipore). Specific antibodies to ALDH1A1 (1:1,000, 611194, BD Bioscience), SAE2 (1:1,000, ab58451, Abcam), SUMO-2,3 (1:500, M114-3, MBL), Ubc9 (1:1,000, #4918, Cell Signaling Technology, Inc.), Oct-1 (1:1,000, #8517, Cell Signaling Technology, Inc.), ubiquitin (1:1,000, MAB1510, Millipore), TRIM21 (1:1,000, sc-25351, Santa Cruz), IRF1 (1:1,000, #8478, Cell Signaling Technology, Inc.), SUMO-1 (1:500, #4930, Cell Signaling Technology, Inc.), SENP2 (1:1,000, sc-67075, Santa Cruz Biotech), PIAS1 (1:1,000, #3550, Cell Signaling Technology, Inc.) and GAPDH (1:1,000, sc-20357, Santa Cruz) were detected using the appropriate secondary antibodies (Licor) and visualized by Odyssey detection system (Licor). The uncropped scans of western blots are provided as Supplementary Fig. 12.

### Protein degradation assay

Oct-1 protein stability was measured on treatment with protein synthesis inhibitor cycloheximide (CHX). Cells treated with 100 μg ml^−1^ CHX (#2112, Cell Signaling Technology, Inc.) were collected at different time points and cell lysate was used for western blot to determine the protein level at different CHX treatment time. Western blot results were quantified by the ImageJ Software (NIH). Three independent experiments were performed and decay curve was plotted.

### Co-IP assay

Cells were lysed in RIPA buffer (50 mM Tris-Cl (pH 8.0), 150 mM NaCl, 1 mM EDTA, 0.5 mM EGTA, 1% Triton X-100, 0.1% sodium deoxycholate and 0.1% SDS) with protease inhibitor cocktail (cOmplet, EDTA-free, Roche), phosphatase inhibitor cocktail (PhosSTOP, Roche) and 20 mM *N*-ethylmaleimide (Sigma). After removal of cell debris by centrifugation, 1 μg of the appropriate antibody and 50 μl Protein G agarose dynabeads (Invitrogen) were added to 500 μg of extracted protein and incubated overnight at 4 °C. Beads were washed three times and boiled with 2 × SDS loading buffer for western blotting. For detecting Oct-1 and TRIM21 interaction, Clean-Blot IP detection kit (#21232 Thermo Fisher Scientific) was used following the manufacture manual to exclude the influence of IgG-fragments heavy chain and light chain.

### Reporter assays

The TRIM21 promoter (−654 to +1,342) luciferase reporter plasmid was a kind gift from Dr Alexander Espinosa (Weill Cornell Medical College). HT29 cells were transfected with TRIM21 promoter luciferase reporter plasmid, pTK-Renilla normalization plasmid (Promega) and empty vector (Ctrl) or SAE2-, UBC9- or SENP1-encoding plasmids and incubated for 48 h. The Dual-Luciferase Reporter Assay System (Promega) was used to quantify luminescence from transfected cells, and normalized results were analysed with two-tailed Student *t*-test.

### Reverse transcription–PCR and real-time quantitative PCR

Total cellular RNA was extracted using RNeasy Mini Kit (Qiagen). Total RNA (2 μg) was reverse-transcribed using Omniscript RT kit (Qiagen) and oligo dT primer. Real-time PCR was performed using the SYBR-Green Master Mix (Applied Biosystems) in a 7900HT instrument (Applied Biosystems). The following primers were used for PCR: Oct-1 sense, 5′-ATGAACAATCCGTCAGAAACCAG-3′; Oct-1 antisense, 5′-GATGGAGATGTCCAAGGAAAGC-3′; TRIM21 sense, 5′-GTCCTGGAAAGGAGTGAGTCC-3′; TRIM21 antisense, 5′-CTGAAAGTATCAGCCACGGATT-3′; GAPDH sense, 5′-AGGTCGGAGTCAACGGATTTG-3′; and GAPDH antisense, 5′-GTGATGGCATGGACTGTGGT-3′.

### Immunohistochemistry

Tumour tissues were fixed in 4% paraformaldehyde, washed with PBS, transferred to 70% ethanol and then embedded in paraffin in accordance with standard procedures. Sections were stained for haematoxylin and eosin, SAE2 (ab58451, Abcam), ALDH1A1 (sc-374076, Santa Cruz), CD44 (#3570, Cell Signaling Technology, Inc.), Oct-1(#8517, Cell Signaling Technnology, Inc.) and TRIM21 (sc-25351, Santa Cruz).

### Mass spectrometry

HCT116 cells were transfected with Flag-tagged Oct-1 (Oct-1) or empty vector (Ctrl) for 2 days. Cell lysates were used for IP with Flag-tag antibody-conjugated M2 beads in a buffer containing Tris-buffered saline, 350 mM NaCl and 0.3% NP40. Binding with Protein A/G agarose beads was performed at 4 °C overnight on a rocking platform, followed by six washes in binding solution containing Tris-buffered saline, 350 mM NaCl and 0.3% NP40. After reduction/alkylation (5 mM dithiothreitol, 30 min, 56 °C; and 25 mM iodoacetamide, in the dark, 20 min), 10 ng trypsin and lysC (modified sequencing grade, Roche) in sodium carbonate 50 mM was added and proteins on beads were incubated overnight at 37 °C while shaking. Then the reaction was stopped with 10 μl 10% formic acid. Peptides were recovered and the beads were removed by filtration through C18 Tips (Proxeon) and elution with 20 μl 50% methanol, 5% formic acid, and subjected to LC–MS/MS sequencing using an LC/MS system consisting of an Eksigent NanoLC Ultra 2D (Dublin, CA) and Thermo Fisher Scientific LTQ Orbitrap XL (San Jose, CA). Protein identifications were made using the commercially available search engine Proteome Discoverer 1.4 (Thermo Fisher Scientific).

### Statistical analyses

For mice experiments, animals were randomly assigned groups for *in vivo* LDA. No animals or samples were excluded from analysis. CSC frequency determinations and the corresponding *P* values in LDA were generated using ELDA software. For other experiments, *P* values were derived using two-tailed Student's *t*-tests from experiments repeated 3–4 times. Estimated variation is indicated as s.d. in each figure. For all graphs, *P<0.05, **P<0.01 and ***P<0.001.

### *In vitro* transcription–translation and ubiquitination assay

DNA fragment coding full-length of Oct-1 was subcloned to the pET28a vector (pET28a-Oct-1). The plasmid was confirmed by DNA sequencing. TNT T7 Quick System Kit (Promega) was used to perform *in vitro* transcription and translation (IVTT) with linearized pET28a-Oct-1. The reaction was incubated at 30 °C for 16 h. Western blot was conducted to determine IVTT efficiency. Reaction mixture without linearized pET28a-Oct-1 was used as control. The *in vitro* transcription and translation product was used as substrate to perform *in vitro* ubiquitination assay. The IVTT product was incubated with 0.05 μM recombinant E1 enzyme UBE1(E-304, Boston Biochem), 0.5 μM recombinant E2 enzyme UBCH5a and UBCH5b (E2-616 and E2-622, Boston Biochem) and 5 μM ubiquitin protein (U-100H, Boston Biochem) with or without addition of 0.5 or 1.0 μg TRIM21 protein (Creatvie-Biomart) and 1 mM ATP in a total volume of 20 μl (30 °C, 16 h). Western blot using anti-Oct-1 and anti-ubiquitin antibodies was performed to detect protein ubiquitination.

### Data availability

All relevant data are available from the authors on request and/or are included with the manuscript (as figure source data or Supplementary Information files).

## Additional information

**How to cite this article:** Du, L. *et al*. Role of SUMO activating enzyme in cancer stem cell maintenance and self-renewal. *Nat. Commun.* 7:12326 doi: 10.1038/ncomms12326 (2016).

## Supplementary Material

Supplementary Information Supplementary Figures 1-12 and Supplementary Tables 1-2

## Figures and Tables

**Table 1:** Protein levels before and after chemoradiation in 18 patient tumour tissues.

SUMO enzyme	Pre-treatment QS*	Post-treatment QS	*P* value
SAE1	4.5±5.7	11.9±3.7	0.0007
SAE2	6.3±4.6	12.2±4.4	0.0003
PIAS1	7.1±2.7	5.5±4.5	0.26

^*^QS, quickscore (staining area multiplied by intensity, possible range of 0–18); the results shown are the average of readings by two independent pathologists.
