# Supplementary material for: Role of SUMO activating enzyme in cancer stem cell maintenance and self-renewal
Source: Nat Commun. 2016 Jul 28;7:12326. doi: 10.1038/ncomms12326 (PMC4974481; doi:10.1038/ncomms12326)
Supplement: Supplementary Information — Supplementary Figures 1-12 and Supplementary Tables 1-2 [file ncomms12326-s1.pdf]

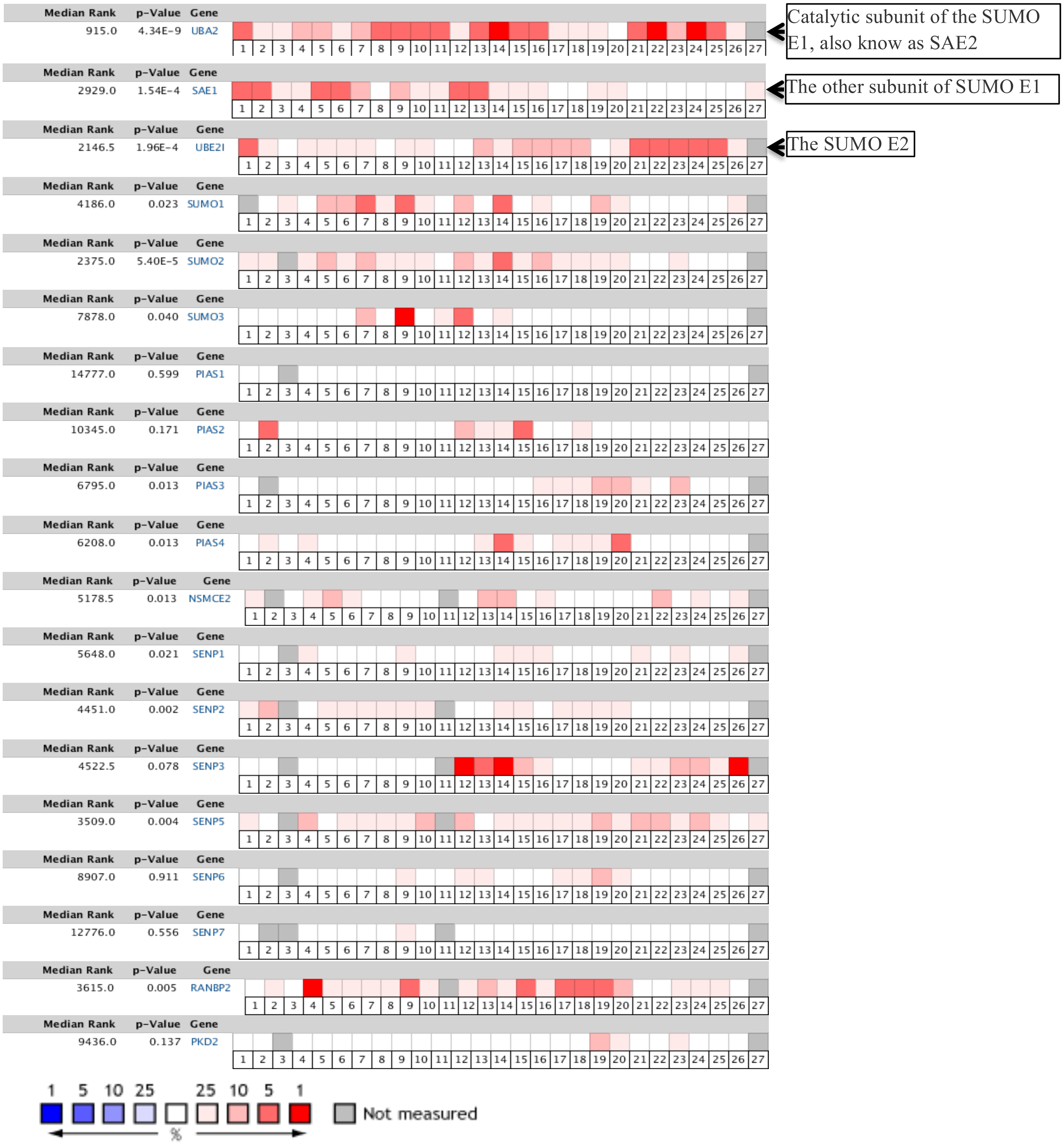

Supplementary Figure 1. Expression of SUMOylation-related genes in colorectal cancer tumor tissues. Genes involved in SUMO modifications were analyzed for in 27 independent datasets of gene expression profiles of colorectal cancer tissues available in Oncomine. The UBA2 (SAE2) gene is consistently among the most overexpressed genes (top 1-10%) across the

27 independent datasets. Red color indicates higher expression and blue color indicates lower expression in cancer tissues than in normal tissues. The 27 datasets are:

1. Rectal Adenocarcinoma vs. Normal *Gaedcke Colorectal, Genes Chromosomes Cancer,2010*

2. Colorectal Adenoma Epithelia vs. Normal *Gaspar Colon, Am J Pathol, 2008*

3. Colorectal Carcinoma vs. Normal *Graudens Colon, Genome Biol, 2006*

4. Colorectal Carcinoma vs. Normal *Hong Colorectal, Clin Exp Metastasis, 2010*

5. Cecum Adenocarcinoma vs. Normal *Kaiser Colon, Genome Biol, 2007*

6. Colon Adenocarcinoma vs. Normal *Kaiser Colon, Genome Biol, 2007*

7. Colon Mucinous Adenocarcinoma vs. Normal *Kaiser Colon, Genome Biol, 2007*

8. Rectal Adenocarcinoma vs. Normal *Kaiser Colon, Genome Biol, 2007*

9. Rectal Mucinous Adenocarcinoma vs. Normal *Kaiser Colon, Genome Biol, 2007*

10. Rectosigmoid Adenocarcinoma vs. Normal *Kaiser Colon, Genome Biol, 2007*

11. Colon Adenocarcinoma vs. Normal *Ki Colon, Int J Cancer, 2007*

12. Skin Squamous Cell Carcinoma vs. Normal *Riker Melanoma, BMC Med Genomics, 2008*

13. Colon Adenoma vs. Normal *Sabates-Bellver Colon, Mol Cancer Res, 2007*

14. Rectal Adenoma vs. Normal *Sabates-Bellver Colon, Mol Cancer Res, 2007*

15. Colorectal Adenocarcinoma vs. Normal *Skrzypczak Colorectal, PLoS One, 2010*

16. Colorectal Carcinoma vs. Normal *Skrzypczak Colorectal, PLoS One, 2010*

17. Colon Adenoma Epithelia vs. Normal *Skrzypczak Colorectal 2, PLoS One, 2010*

18. Colon Adenoma vs. Normal *Skrzypczak Colorectal 2, PLoS One, 2010*

19. Colon Carcinoma Epithelia vs. Normal *Skrzypczak Colorectal 2, PLoS One, 2010*

20. Colon Carcinoma vs. Normal *Skrzypczak Colorectal 2, PLoS One, 2010*

21. Cecum Adenocarcinoma vs. Normal *TCGA Colorectal, No Associated Paper, 2011*

22. Colon Adenocarcinoma vs. Normal *TCGA Colorectal, No Associated Paper, 2011*

23. Colon Mucinous Adenocarcinoma vs. Normal *TCGA Colorectal, No Associated Paper, 2011*

24. Rectal Adenocarcinoma vs. Normal *TCGA Colorectal, No Associated Paper, 2011*

25. Rectal Mucinous Adenocarcinoma vs. Normal *TCGA Colorectal, No Associated Paper, 2011*

26. Rectosigmoid Adenocarcinoma vs. Normal *TCGA Colorectal, No Associated Paper, 2011*

27. Colon Carcinoma vs. Normal *Zou Colon, Oncogene, 2002*

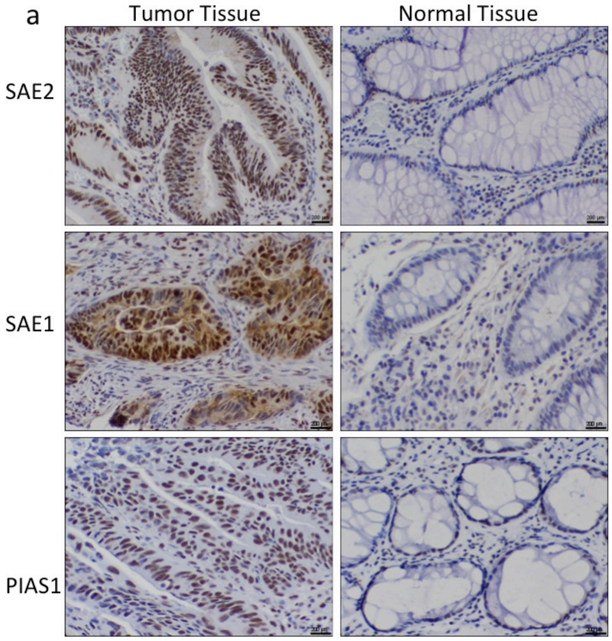

b

Protein	Colorectal Cancer Tissue	Matched Normal Tissue
SAE1	65%	7%
SAE2	53%	0%
PIAS1	10%	0%

Supplementary Figure 2. IHC staining of colorectal cancer tissue and matched normal control with SAE2, SAE1 and PIAS1 antibodies. (a) Representative IHC staining of SAE2 (top panel), SAE1 (middle panel) and PIAS1 (bottom panel) in colorectal cancer specimens (left panel) and matched normal tissues (right panel). Scale bar presents 200 μm. (b) Statistical analysis of the percentage of tissue showing high protein levels from IHC staining results. Tumor specimens from 51 patients with colorectal cancer were examined for the expression of SAE2, SAE1 and PIAS1 in paired benign and malignant tissues by IHC staining. Percentage of tissues showing high IHC staining in 51 colorectal cancer specimens are shown.

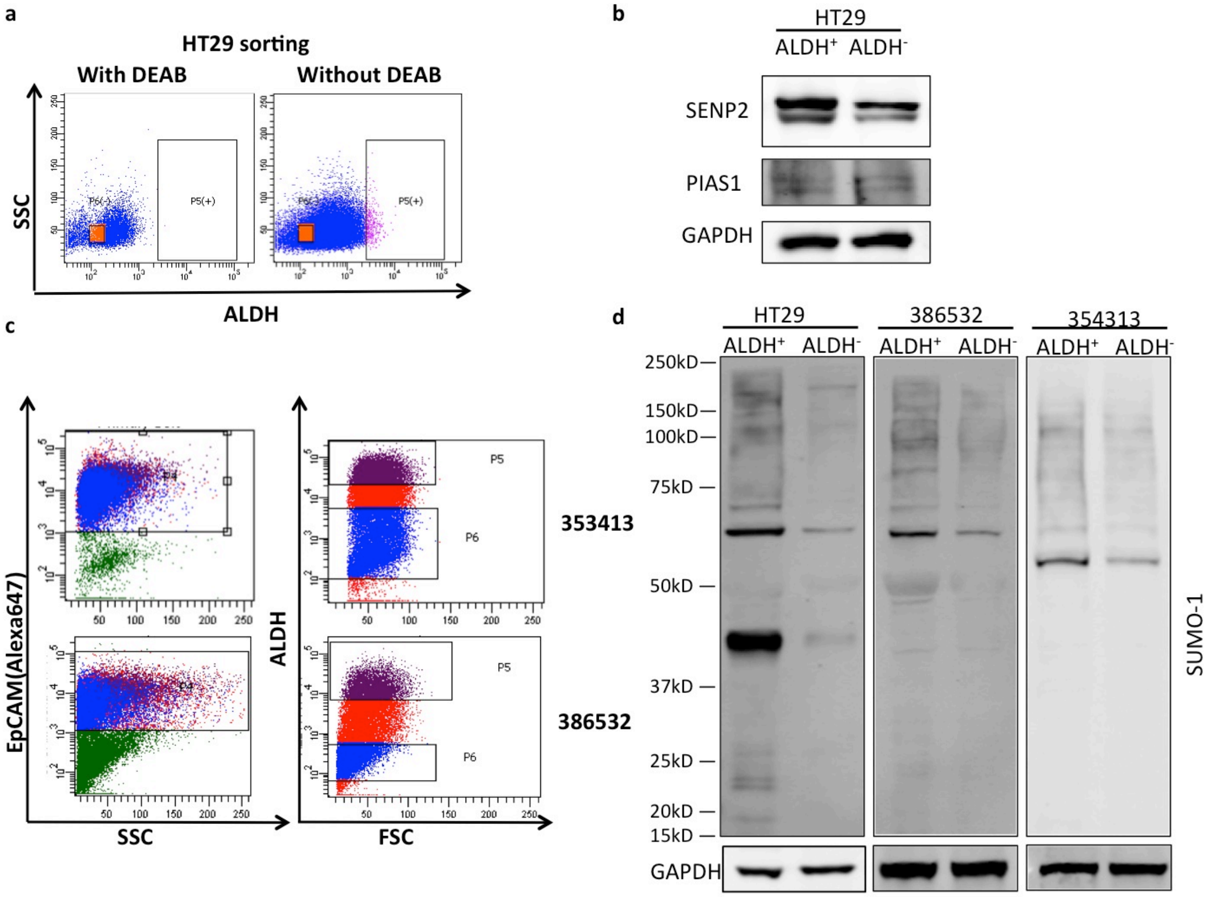

Supplementary Figure 3. ALDH$^+$ human colorectal cancer cells have higher global SUMOylation levels. (a) Representative Aldefluor sorting of HT-29 cells. Control samples incubated with the inhibitor, DEAB, were used to ensure identification of ALDH$^+$ (boxed region) and ALDH$^-$ cells. (b) Representative Western blot of SENP2, PIAS1 and GAPDH of ALDH$^+$ and ALDH$^-$ cells isolated from HT29. (c) Aldefluor and EpCAM sorting of PDX colorectal cancer primary cultures (354313 and 386532). EpCAM (Alexa-647) was used to isolate colorectal cancer cells from stroma or fibrablasts (EpCAM$^+$ cells labeled as P4), and ALDH$^+$ (P5) and ALDH$^-$ cells (P6) were sorted from EpCAM$^+$ population using Aldefluor assay kit. (d) Representative Western blot of SUMO-1 in ALDH$^+$ and ALDH$^-$ cells isolated from HT29 and primary PDX culture; GAPDH, loading control.

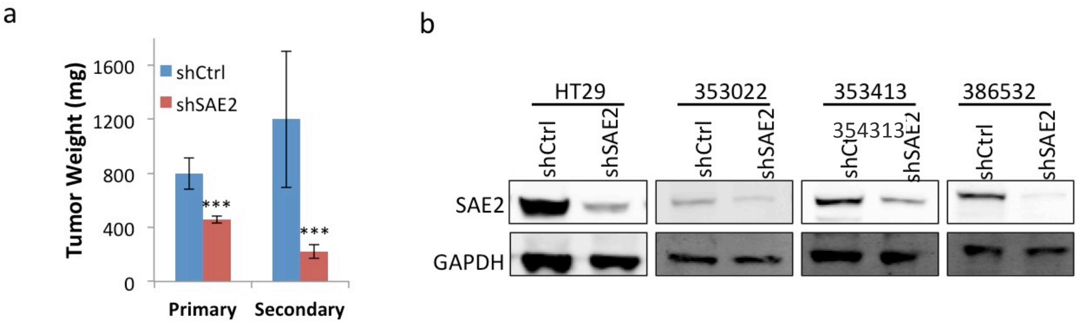

c

Group			Spheroid containing wells/total wells, cells/well 1000	100	10	1	Spheroid-initiating Cell Frequency Estimate (95% confidence interval)	Fold	*p*-value
HT29	Primary	shCtrl	24/24	24/24	20/96	3/96	1/35 (1/26-1/48)	1.000	
		shSAE2	24/24	24/24	6/96	1/96	1/62(1/44-1/88)	0.565	0.0256
	Secondary	shCtrl	24/24	24/40	22/128	9/128	1/70 (1/50-1/93)	1.000	
		shSAE2	24/24	12/40	10/128	1/128	1/198(1/136-1/290)	0.353	7.25E-06
353022	Primary	shCtrl	24/24	54/56	10/96	4/96	1/44 (1/34-1/57)	1.000	
		shSAE2	24/24	50/56	2/96	1/96	1/67(1/51-1/89)	0.650	0.035
	Secondary	shCtrl	24/24	53/56	24/128	10/128	1/36.5 (1/28.7-1/46.6)	1.000	
		shSAE2	24/24	48/56	9/128	1/128	1/69.5(1/53.5-1/90.3)	0.525	5E-04
354313	Primary	shCtrl	68/68	110/131	19/136	1/136	1/58 (1/48.2-1/70)	1.000	
		shSAE2	62/63	81/132	12/132	0/136	1/118(1/95.7-1/147)	0.492	4.77E-07
	Secondary	shCtrl	56/56	120/120	40/136	2/142	1/25.7 (1/20.9-1/31.6)	1.000	
		shSAE2	27/27	116/120	12/120	0/138	1/42.4(1/34.9-1/51.6)	0.606	0.00107
386532	Primary	shCtrl	48/48	80/96	56/144	5/152	1/38.1 (1/31-1/46.8)	1.000	
		shSAE2	48/48	42/136	11/144	0/144	1/231.4(1/181.8-1/294.5)	0.165	3.18E-32
	Secondary	shCtrl	96/96	96/96	30/120	4/144	1/27.8 (1/22.3-1/34.7)	1.000	
		shSAE2	96/96	95/96	7/128	1/144	1/45.1(1/36.6-1/55.6)	0.616	0.004

Supplementary Figure 4. Additional supporting data for LDA of Fig. 2. (a) Tumor weight of *in vivo* LDA of shCtrl and shSAE2 group implanted with 1,000 cells/mouse (corresponding to Fig. 2a and 2b). (b) Representative Western blot for SAE2 expression in HT29 cells or primary culture of colorectal PDX tissues 353022, 354313 and 386532 showing SAE2 knockdown; GAPDH, loading control. (c) Detailed data of *in vitro* LDA. HT29 and primary culture of colorectal cancer PDX (353022, 354313 and 386532) cells were transduced with control (shCtrl) or SAE2-targeting shRNA (shSAE2). Then the cells were dissociated after primary spheres were formed and the dissociated cells were plated for LDA. The percentage of wells with spheres was determined for primary and secondary LDA. CSC frequency (sphere formation cells) and *p*-values were calculated as described in Methods.

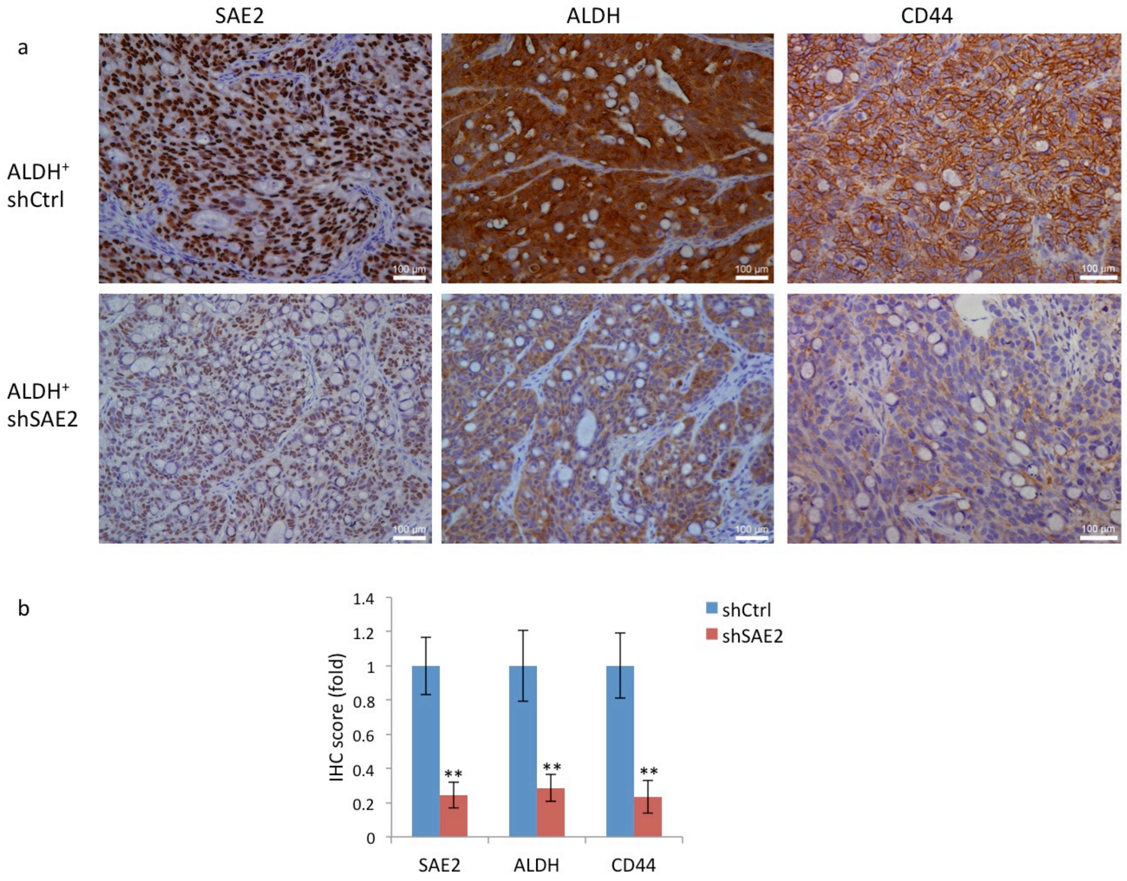

Supplementary Figure 5. SAE2 knockdown in ALDH$^+$ cells reduced SAE2, ALDH1A1 and CD44 levels in xenograft tumor tissue. (a) IHC staining of tumor tissues from *in vivo* LDA with or without SAE2 knockdown in HT29 ALDH$^+$ cells (Fig. 3). The scale bar represents 100 μm. (b) Statistical analysis of IHC staining. 10 slides of each group were scored for staining intensity (range from 0-no staining, 1-weak, 2-intermediate, 3-strong intensity) multiplied by the percentage of the staining area (0-100); range = 0-300. Two-tailed Student *t*-test was used to determine *p* values (** $p < 0.01$).

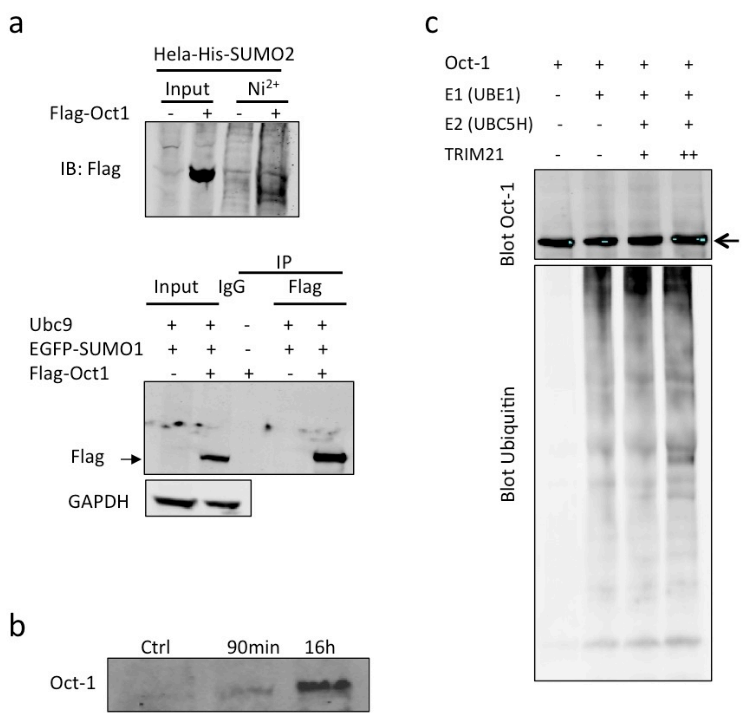

Supplementary Figure 6. Oct-1 SUMOylation and ubiquitination assays. (a) Upper panel, HeLa cells stably expressing His-SUMO2 were transfected with or without Flag-tagged Oct-1. After 2 days, cells were collected and SUMO-2 modified proteins were pull-down by Ni-NTA affinity chromatogaphy under denaturing condition, followed by Western blotting with Flag antibody. No SUMOylated-Oct-1 band was detected. Lower panel, Flag-tagged Oct-1 was co-transfected with UBC9 and EGFP-SUMO1 in 293T cells. Cell lysate was immunopecipitated and western blotted with Flag antibody. Only unmodified Oct-1 band was detected. (b) Representative blot of *in vitro* transcription and translation (IVTT) of Oct-1 using different incubation times. DNA encoding full-length Oct-1 was subcloned into pET28a vector. TNT T7 Quick System Kit (Promega) was used to perform IVTT with linearized pET28a-Oct-1. Oct-1 was detected by Western blot. Reaction mixture without linearized pET28a-Oct-1 was used as control "Ctrl". (c) Representative blot of *in vitro* ubiquitination assay with IVTT Oct-1 by incubating with recombinant E1 (UBE1), E2 enzyme (UBCH5), and ubiquitin, and without or with 0.5 or 10 µg TRIM21 protein (obtained from Creative-Biomart). Oct-1 and ubiquitin were detected by Western blot using anti-Oct-1 and anti-ubiquitin antibodies.

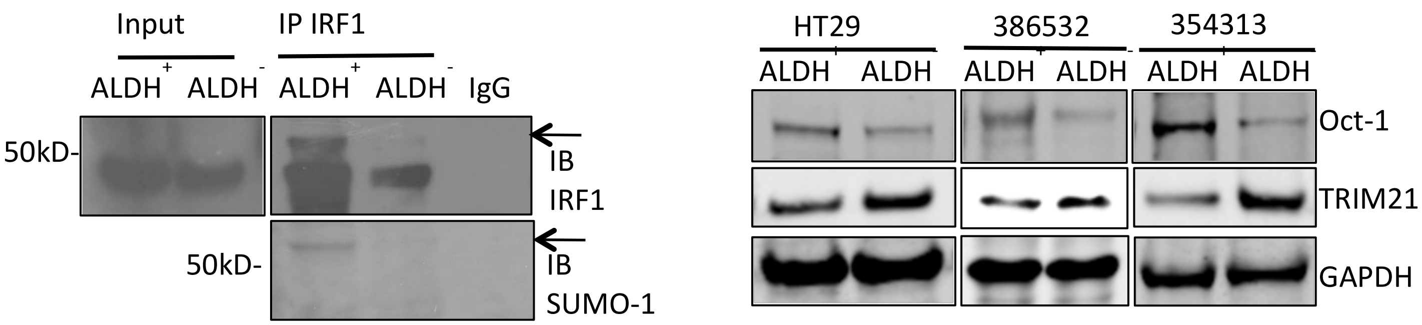

Supplementary Figure 7. **Additional data showing connection of SUMOylated IRF1, Oct1 and TRIM21.** (a) SUMOylated IRF1 was only present in HT29 ALDH$^+$ cells but not in ALDH$^-$ cells. HT29 ALDH$^+$ and ALDH$^-$ cell lysate were used for IP with anti-IRF1 antibody and protein G beads. Equal amount of ALDH$^+$ cell lysate was incubated with rabbit IgG as blank control. Clean-Blot IP detection kit was used for western blotting of IRF1 (upper panel) and SUMO-1 (bottom panel). SUMO-modified IRF1 was only observed in ALDH$^+$ lysates and is indicated by arrows. (b) ALDH$^+$ cells have higher Oct-1 level and lower TRIM21 level than ALDH$^-$ cells. Western blot shows Oct-1 and TRIM21 level of ALDH$^+$ and ALDH$^-$ cells isolated from colorectal cancer cell line HT29 and primary culture of colon cancer PDX tumor tissue (386532 and 354313 stands for PDX tissue from different patient specimen).

Group		Spheroid containing wells/total wells (cells/well)					Spheroid-initiating Cell Frequency	Fold	*p*-value	
		**5000**	**1000**	**100**	**10**	**1**	Estimate (95% confidence interval)		**a**	**b**
**Primary**	**shCtrl+EV**	64/64	80/80	128/128	32/128	2/136	1/28(1/23-1/34)	1.000		
	**shSAE2+EV**	64/64	88/88	114/128	7/128	0/128	1/57(1/48-1/69)	0.481	6.71E-07	
	**shSAE2+Oct-1**	64/64	88/88	106/112	23/120	1/136	1/39(1/32-1/48)	0.701	0.018	0.0105
**Secondary**	**shCtrl+EV**	48/48	96/96	119/120	27/120	2/96	1/29.8(1/24.2-1/36.7)	1.000		
	**shSAE2+EV**	72/72	96/96	96/120	6/120	0/96	1/73(1/59.7-1/89.3)	0.408	2.70E-09	
	**shSAE2+Oct-1**	56/56	96/96	115/120	17/120	1/96	1/40(1/32.8-1/48.8)	0.745	0.0535	5.11E-05

Supplementary Figure 8. Detailed data showing that overexpression of Oct-1 partially restored CSC population in HT29-shSAE2 cells. Stable cell lines were generated with lentivirus expressing plentiCMV-hygro empty vector (EV) or Flag-Oct-1 in HT29 shCtrl and shSAE2 cells. Spheroid-formation assay was carried out with limited dilution series. CSC frequency and *p*-value were determined as described in Methods. **a**, *p*-value calculated by comparing shSAE2+Oct-1 and shSAE2+EV cells with shCtrl+EV; **b**, *p*-value calculated from comparison between shSAE2+EV and shSAE2+Oct-1 cells.

a

Group		Spheroid containing wells/total wells cells/well				Spheroid-initiating Cell Frequency	Fold	*p*-value
		1000	100	10	1	Estimate (95% confidence interval)		
Primary	shCtrl	72/72	96/96	38/104	6/112	1/20.5 (1/16.1-1/26.1)	1	
	shUBC9#1	84/84	128/128	9/120	1/120	1/37.3(1/30.7-1/45.3)	0.55	0.000344
	shUBC9#2	96/96	119/120	6/120	0/136	1/41.8(1/34.3-1/50.8)	0.49	2.10E-05
Secondary	shCtrl	72/72	96/96	52/104	12/112	1/13.8 (1/10.9-1/17.5)	1	
	shUBC9#1	96/96	128/128	26/120	2/120	1/30.2(1/24.6-1/37)	0.46	2.48E-06
	shUBC9#2	96/96	119/120	17/120	0/136	1/35.8(1/29.4-1/43.8)	0.39	1.04E-08

b

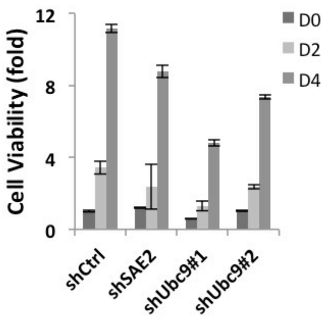

Supplementary Figure 9. UBC9 knockdown also reduced CSC frequency and self-renewal as determined by *in vitro* LDA as well as proliferation. (a) Stable Ubc9 knockdown cell lines were generated with two independent shRNAs (shUBC9#1 and shUBC9#2) by lentiviral transduction of HT29 cells. shCtrl, shUBC9#1 and shUBC9#2 cells were dissociated after primary spheres were formed and dissociated cells were plated for LDA. CSC frequency and *p*-values were calculated as described in Methods. (b) Knockdown of SAE2 or UBC9 also suppressed cell proliferation. Cell viability was measured for stable cell lines shCtrl, shSAE2, shUBC9#1 and shUBC9#2 on day 0 (D0), day 2 (D2) and day 4 (D4) after cell seeding using CellTiter-Glo (Promega, Inc). Results were normalized to shCtrl at D0.

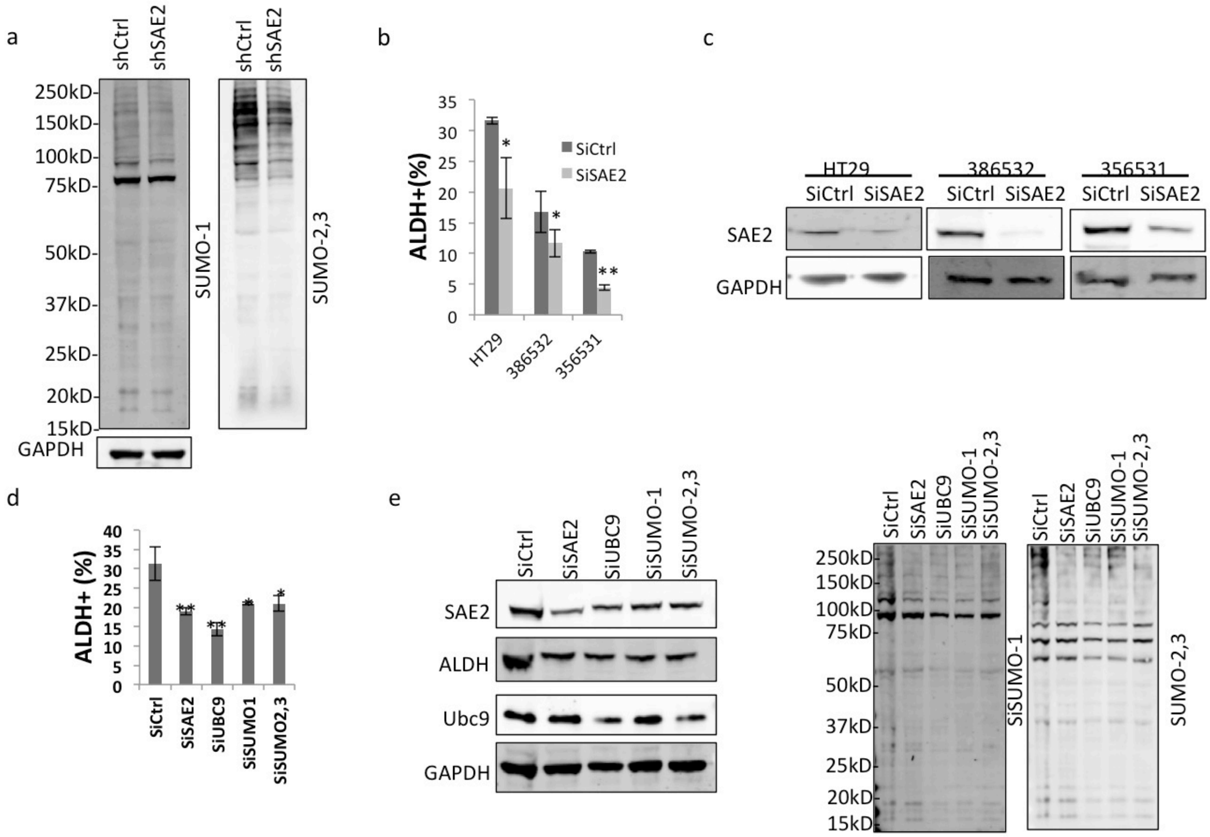

Supplementary Figure 10. Inhibition of SUMOylation reduced colorectal cancer cell ALDH$^+$ population. (a) Representative Western Blot of global SUMOylation (SUMO-1 and SUMO-2,3) in stable HT29 cell line expressing control non-silencing shRNA (shCtrl) or SAE2-targeting shRNA (shSAE2); GAPDH, loading control. (b) Transient knockdown of SAE2 with siRNA transfection reduced ALDH$^+$ population in HT29 and primary colorectal cancer PDX cultures (386532 and 356531) as determined by FACS Aldefluor assay. Three independent knockdown experiments were performed and results were analyzed with two-tailed Student's *t*-test. (c) Representative Western blot of SAE2 expression of samples in (b); GAPDH, loading control. (d) Transient knockdown of SAE2, UBC9, SUMO-1 or SUMO-2,3 with siRNA transfection reduced ALDH$^+$ population in HT29 cells as determined by FACS Aldefluor assay. (e) Representative Western blot of SAE2, ALDH, UBC9, SUMO-1 and SUMO-2,3 of samples in (d) ; GAPDH, loading control. (* $p < 0.05$, ** $p < 0.01$)

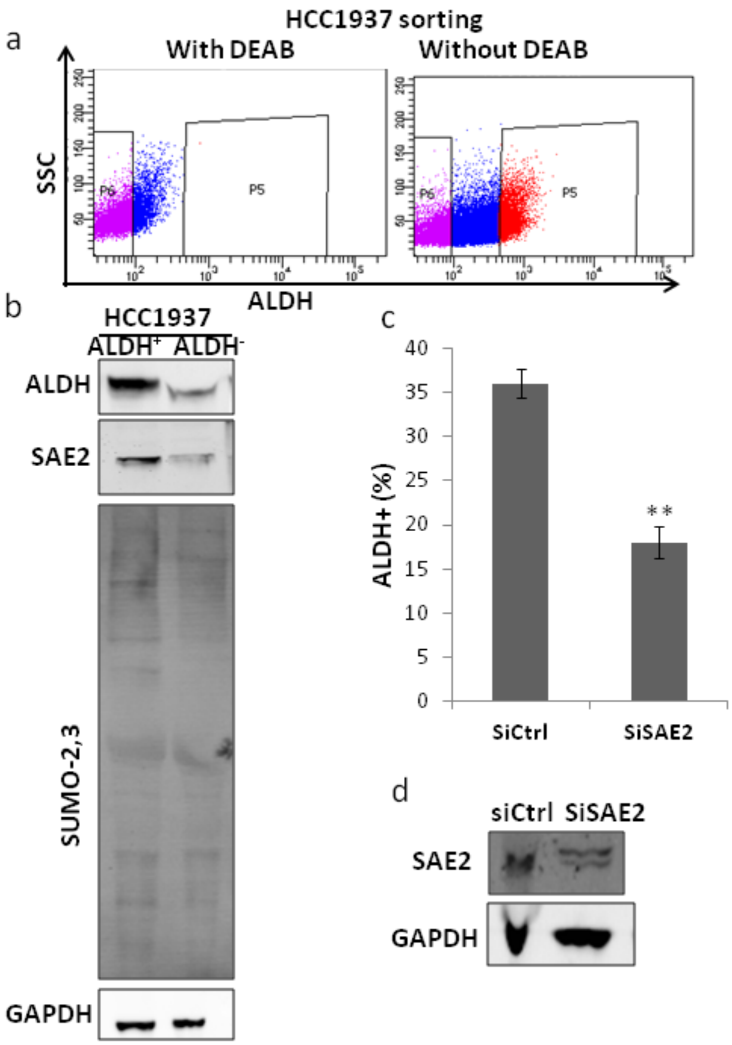

Supplementary Figure 11. ALDH^{+} cells isolated from the breast cancer cell line HCC1937 also have higher SAE2 and global SUMOylation levels. (a) Representative Aldefluor sorting of HCC1937 cells. Control samples incubated with the inhibitor, DEAB, were used to ensure the identification of ALDH^{+} (boxed region P5) and ALDH^{-} cells (boxed region P4). (b) Representative Western blot of ALDH, SAE2, and SUMO-2,3 of ALDH^{+} and ALDH^{-} isolated from HCC1937 cells; GAPDH, loading control. (c) Knockdown of SAE2 reduced ALDH^{+} population in HCC1937 cells (**, $p < 0.01$). (d) Western blot of SAE2 of the FACS samples shown in (c).

Supplementary Figure 12. All original blots in main figures.

Fig.1d

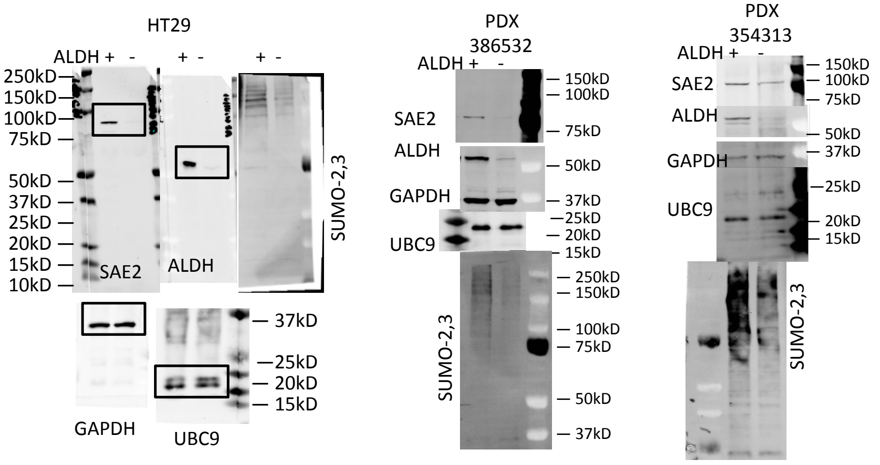

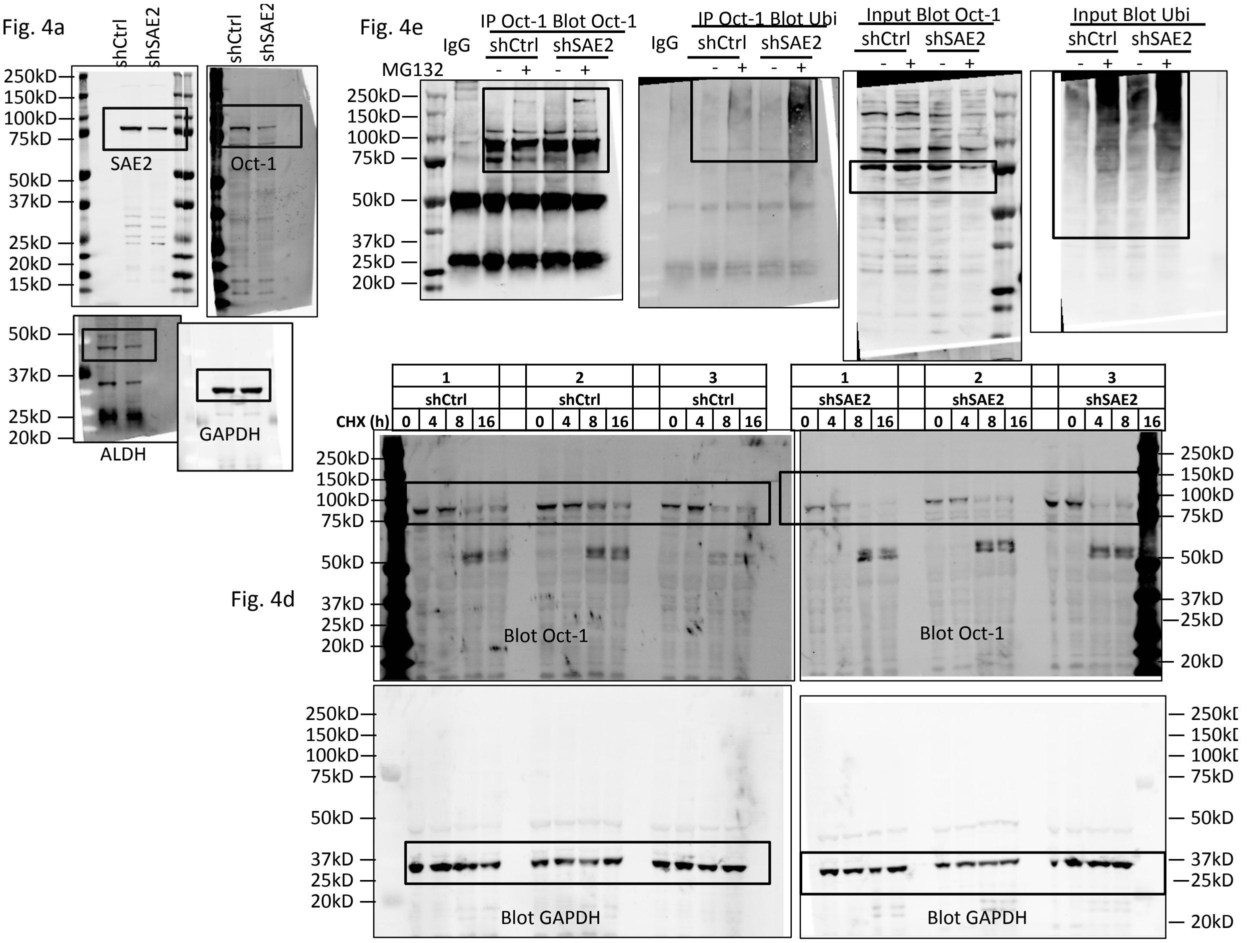

Fig.5b

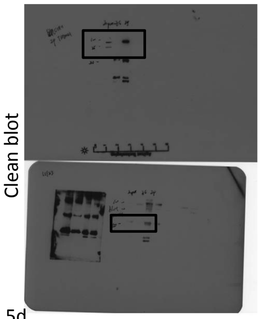

Fig. 5c

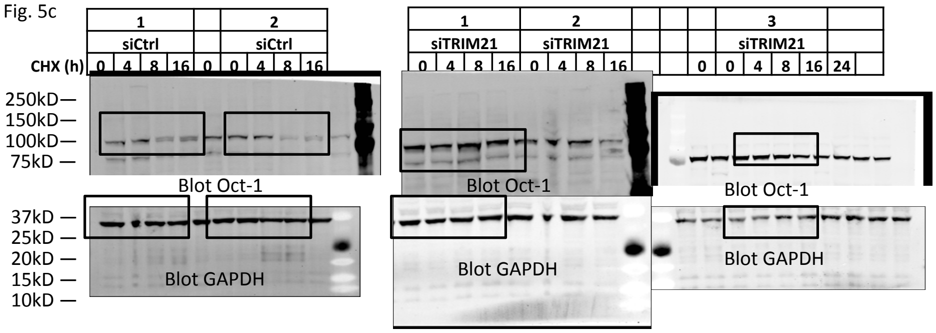

Fig.5d

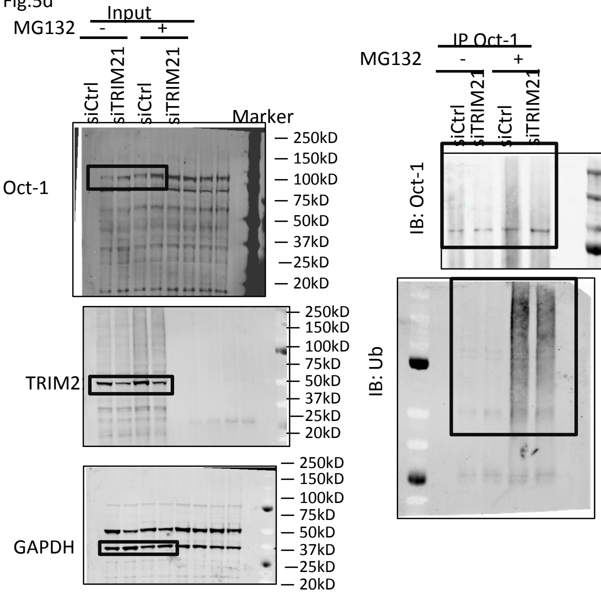

Fig.5e

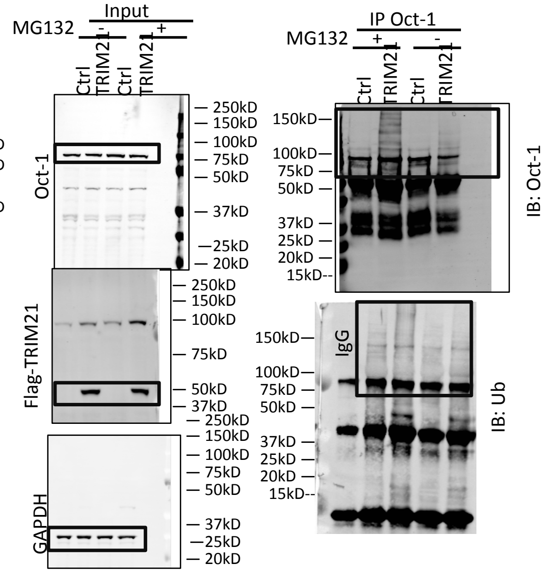

Fig.6b

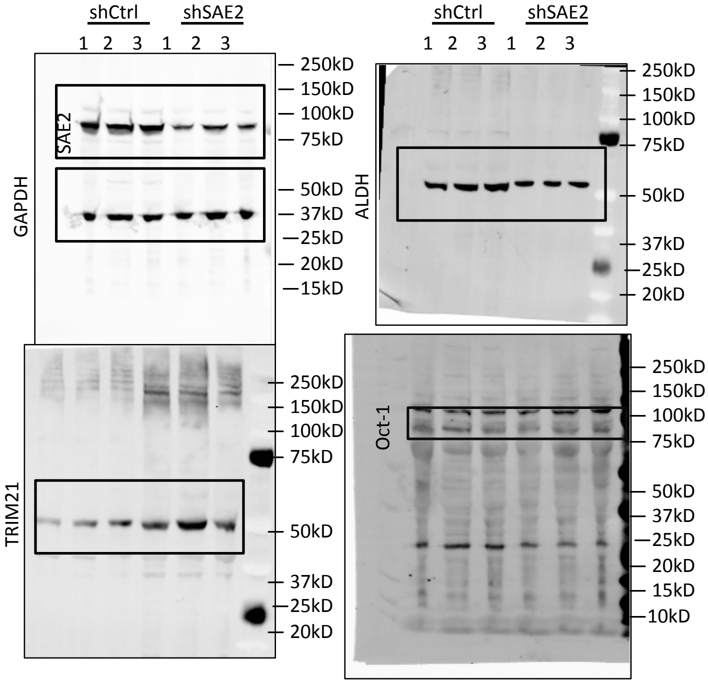

Fig.6f

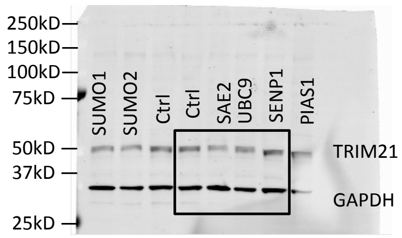

Fig6g

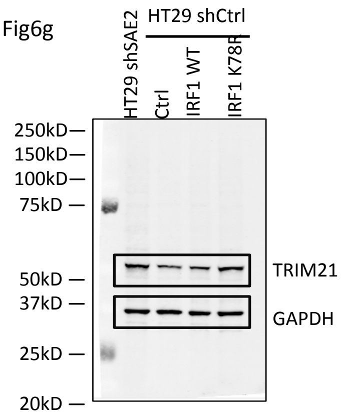

Fig7b

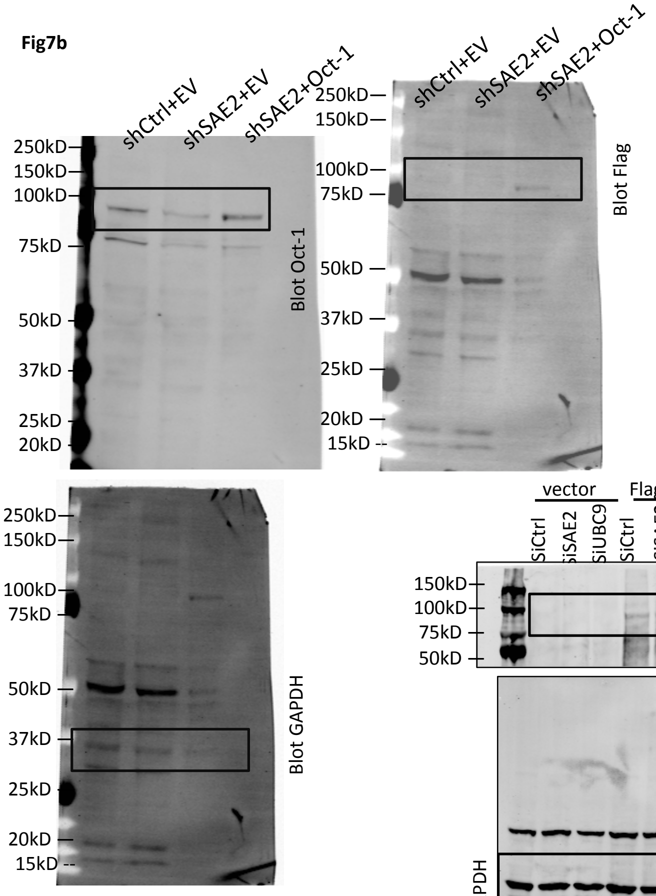

shCtrl+EV
shSAE2+EV
shSAE2+Oct-1
250kD
150kD
100kD
75kD
50kD
37kD
25kD
20kD
15kD
Blot Oct-1
Blot Flag
Blot GAPDH

Fig7d

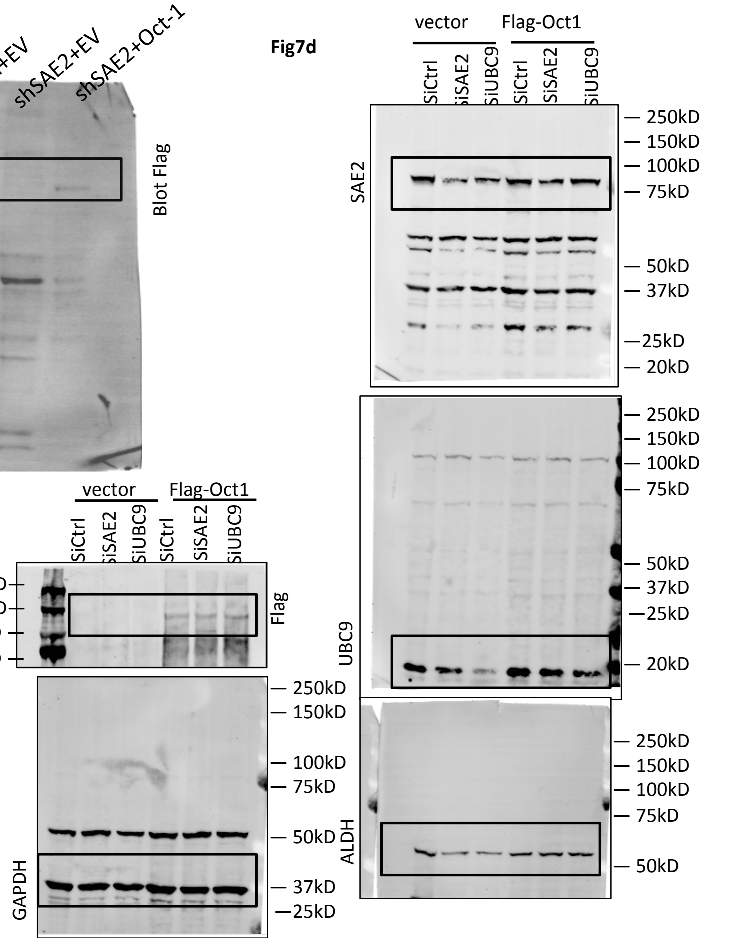

vector
Flag-Oct1
SiCtrl
SiSAE2
SiUBC9
SAE2
UBC9
ALDH
Flag
GAPDH
250kD
150kD
100kD
75kD
50kD
37kD
25kD
20kD

Supplementary Table 1: mRNA gene expression level of SUMOylation-related genes in colorectal cancer cell lines compared with normal colonic mucosa

Gene	isoform count	HCT116 (RPKM[1])	HT29 (RPKM)	Average Control (RPKM)	HCT116 vs. Control	HT29 vs. Control
**SAE2 (UBA2)**	**1**	**60.09**	**91.02**	**6.13**	**9.80**	**14.85**
**SAE1**	**4**	**103.75**	**108.24**	**17.34**	**5.98**	**6.24**
SUMO1	3	61.64	41.83	10.67	5.78	3.92
SUMO2	2	97.61	80.04	20.41	4.78	3.92
RANBP2	1	12.81	9.85	2.79	4.58	3.52
SENP3	1	21.15	29.76	9.15	4.47	4.94
PIAS2	2	9.36	3.88	2.11	4.43	1.83
PIAS3	1	6.64	7.95	1.53	4.34	5.20
NSMCE2 (Mms21)	1	21.78	33.85	5.13	4.24	6.59
Ubc9 (E2, UBE2I)	4	113.94	101.28	29.31	3.89	3.46
SENP2	1	11.60	11.82	3.18	3.66	3.68
PKD2 (Pc2)	1	3.16	1.94	0.89	3.56	2.18
SENP6	2	8.96	3.50	1.41	3.32	2.17
SENP5	1	7.41	6.05	1.88	3.07	2.82
SENP1	1	5.93	6.81	1.16	2.79	2.97
SENP7	2	2.28	0.78	0.34	1.72	0.83
PIAS1	1	9.87	17.31	6.79	1.45	2.55
PIAS4	1	5.09	9.23	6.52	0.78	1.42
SUMO3	1	19.74	67.49	30.72	0.64	2.20
GAPDH	1	2100.40	2313.06	1094.17	1.92	2.11
TUBA4A (α-tubulin)	1	12.05	118.52	33.62	0.36	3.53
ACTB(β-actin)	1	528.15	1692.28	2300.95	0.23	0.74

[1]RPKM = reads per kilobase of exon per million mapped reads

Supplementary Table 2. Oct1-interacting proteins identified by co-IP and mass spectrometry analysis.

Accession	Protein	Score[1]	Coverage[2]	MW [kDa]
B4DY30	PRMT5	57.62	18.72	67.6
B4E0B3	Histone H2A	39.21	17.12	12.1
P11142	HSP70	36.82	12.69	70.9
B4DNE0	EF1a	34.95	15.19	42.6
Q8WU19	TUBULIN	33.99	15.22	37.2
14249924	XRCC6	28.05	11.82	69.8
P11021	GRP78	25.12	4.28	72.3
Q45KI0	Trypsin I	21.80	11.90	9.2
P07900	HSP90	20.91	7.51	84.6
**P19474**	**TRIM21**	**19.87**	**5.47**	**54.1**
Q59GP5	eEF1a2	18.71	14.12	36.9
Q5T8M8	ACTIN	13.93	11.50	32.0
Q969I0	KRT8	13.86	11.78	41.1
Q6AI40	DKFZ	13.51	29.85	7.2
Q6P3W7	SCYL2	11.71	3.12	103.6
10863945	XRCC5	11.63	3.14	82.7
Q5T6W2	HNRNPK	11.29	11.87	41.8
B2R4V4	BANF1	9.35	15.73	10.0
Q5JUV3	NT5C2	8.61	7.47	20.4
B0QYB2	KCTD17	7.23	20.63	7.0
Q0QEN7	ATP5B	7.09	3.15	48.1
E9PCY7	HNRNPH1	6.75	6.53	47.1
1698399	BRCA1	6.25	2.42	207.6
Q96BA7	HNRPU	6.16	3.05	79.7
Q96HS1	PGAM5	6.14	4.15	32.0
Q96IR1	RPS4X	5.56	6.58	27.2
C9J5S7	PPIA	5.16	11.57	13.2
Q96CJ1	EAF2	4.71	13.08	28.8
Q8WUW7	PKM2	4.32	4.96	37.3
331284178	NCOR2	4.12	1.03	273.5
P78333	GPC5	3.84	2.27	63.7
Q9NZ81	PRR13	3.74	22.30	15.4
Q99714	HSD17B10	3.66	7.66	26.9
C9JYQ9	RPL22L1	3.55	10.74	14.5
P39019	RPS19	3.48	8.97	16.1
C9J4Z3	RPL37A	3.36	26.47	7.6
Q76N53	RPL31	3.00	29.79	5.6
P16422	EPCAM	2.95	5.10	34.9
E5RH77	RPS14	2.83	11.19	14.3
Q5T0P8	RPS24	2.63	11.54	15.1
119623829	TNF2	2.41	7.73	25.6
85681283	FDXR	2.31	2.44	53.8
78070398	ATF2	2.07	3.58	48.0
21618340	STAT3	2.06	1.17	88.0

[1] Score – the sum of the scores of the individual peptides

[2] Coverage – the percentage of the protein sequence covered by identified peptides

Blue-labeled proteins have been identified as Oct-1 interacting proteins in previous studies.

Red-labeled protein TRIM21, ubiquitin E3 ligase.
